# *In utero* and lactational exposure to low-doses of the pyrethroid insecticide cypermethrin leads to neurodevelopmental defects in male mice—An ethological and transcriptomic study

**DOI:** 10.1371/journal.pone.0184475

**Published:** 2017-10-11

**Authors:** Anthony Laugeray, Ameziane Herzine, Olivier Perche, Olivier Richard, Céline Montecot-Dubourg, Arnaud Menuet, Séverine Mazaud-Guittot, Laurianne Lesné, Bernard Jegou, Stéphane Mortaud

**Affiliations:** 1 Immunologie et Neurogénétique Expérimentales et Moléculaires – UMR7355 CNRS – Orléans, France; 2 Département de génétique, Center Hospitalier Régional, Orléans, France; 3 IRSET INSERM U 1085, Université de Rennes I, Rennes, France; Indian Institute of Toxicology Research, INDIA

## Abstract

Accumulating evidence suggests that developmental exposure to environmental chemicals may modify the course of brain development, ultimately leading to neuropsychiatric / neurodegenerative disorders later in life. In the present study, we assessed the impact of one of the most frequently used pesticides in both residential and agricultural applications − the synthetic pyrethroid cypermethrin (CYP) − on developmental neurotoxicity (DNT). Female mice were perinatally exposed to low doses of CYP (5 and 20 mg/kg body weight) from gestation to postnatal day 15. Behavioral analyses were performed during the offspring’s early life and during adulthood. Postnatal analyses revealed that perinatal exposure to CYP disturbed motor development without modifying sensory and communicative skills. We found that later in life, CYP-exposed offspring expressed maladaptive behaviors in response to highly challenging tasks and abnormal sociability. Transcriptomic analyses performed in the offspring’s brain at the end of the exposure, highlighted mitochondrial dysfunction as a relevant pathomechanism underlying CYP-induced DNT. Interestingly, several genes involved in proteostasis maintenance were also shown to be dysregulated suggesting that alterations in biogenesis, folding, trafficking and degradation of proteins may significantly contribute to CYP-related DNT. From a regulatory perspective, this study highlights that behavioral and transcriptomic analyses are complementary tools providing useful direction for better DNT characterization, and as such, should be used together more systematically.

## Introduction

Pre and postnatal periods are vital time spans for brain development. This is when the production, migration and proliferation of neurons are achieved, and the elementary structure of the brain is built. Disturbances of these tightly regulated processes may negatively impact the establishment of basic neuronal circuits, weakening the fundamental structure of the brain. Such modifications may also be responsible for permanent impairments, thereby leading to a wide range of enduring adverse impacts on emotional cognition later in life. These core principles are the basis for the Barker’s hypothesis, which is better known as the DOHaD approach, *i*.*e*. the Developmental Origins of Health and Diseases [1]. According to this concept, the susceptibility to some adult-onset diseases may originate from *in utero* and neonatal environmental exposures. Some of these chemicals are produced in our bodies while others enter through contaminants in the air we breathe, the water we drink and the food we eat.

Among the contaminants that are likely to disturb brain development, cypermethrin (CYP) and other pyrethroid insecticides are highly relevant candidates as the general population is extensively exposed to this family of pollutants through dietary intake and residential application in gardens and homes for pest-control purposes [2, 3]. CYP, and more generally, pyrethroids, are included in over 3,500 registered products widely used in households including on pets, as treatment for clothing, mosquito control, and agriculture. While the use of pyrethroids has increased during the past decade, the use of organophosphate compounds has considerably decreased because of their inherent toxicity. All the pyrethroids, including the type II compound CYP, primarily act by disrupting the function of voltage-sensitive sodium channels (VSSC). They not only slow the opening of VSSCs but also slow their rate of closing. As a result, sodium channels open after smaller depolarization and remain open longer, allowing more sodium ions to cross and depolarize the neuronal membrane [4]. Epidemiological studies have pointed out that pyrethroid exposure during pregnancy has resulted in developmental neurotoxicity as evidenced by a delayed neurocognitive development in 6 year old children [5]. Laboratory studies confirmed this data as exposure to pyrethroids during the “brain growth spurt period” and this has been shown to induce long-term irreversible changes such as delayed mental development with reduced motor functions, visual acuity, reduced short-term memory, and attention deficit [6, 7]. Even if animal studies allowed for a better understanding of the impact of pyrethroids on development, reproduction and adult toxicity, the potential for developmental neurotoxicity of pyrethroids is not well understood. This has been demonstrated by the limited number of reported studies [8]. Indeed, only 1 study for permethrin, 1 study for cyfluthrin and 3 studies for CYP and fenvalerate have been conducted to investigate their role of developmental neurotoxicity in animals [8]. For some years, several authors have claimed that further research is needed to clarify the possible risks associated with long-term environmental exposure to pyrethroids [9, 10]. In line with this, most animal studies addressing the developmental neurotoxicity of pyrethroids have not complied with the latest guidelines (OECD 426) for Developmental Neurotoxicity Testing (DNT) issued by the European Organization for Economic Cooperation and Development [11]. In addition, only the oral route of exposure was assessed in these studies despite the relevance of another route of exposure, through airway inhalation. In support of this, we recently demonstrated that when the herbicide, glufosinate ammonium was perinatally administered through the intranasal route, the neurodevelopmental outcomes were strikingly reminiscent of ASD-like symptoms in exposed offspring, even at doses largely below the current NOAEL in mice [12, 13]. These results suggest that airway exposure has to be addressed more systematically during regulatory testing procedures, especially when dealing with airway environmental toxicants such as pyrethroids.

With the aim of providing updated data, aligned with guideline OECD 426, on CYP developmental neurotoxicity, female mice were intranasally exposed during pre and postnatal periods to CYP and neurodevelopmental endpoints and whole brain transcriptomic changes and long-lasting neurobehavioral changes were assessed in male offspring. In keeping with this objective, we were also interested in raising the awareness of an alternative way to analyze the behavior of mice to get more accurate data. This approach was based on an eco-ethological appraisal of contexts to which mice are confronted when tested in classical apparatuses such as the forced swim task or the elevated plus maze. The behavioral response emitted by animals in these tasks was dynamic and subsequently deserves to be interpreted as such. A similar approach has been used in the openfield and has been shown to provide more reliable data than the classical method [14]. This method relies on the concept of emotional cognition stating that stress, anxiety, and other emotions are key elements of cognition and include selective attention, working memory, and cognitive control. In turn, circuits involved in attention, executive control, and working memory contribute to the regulation of emotion. This means that the distinction between the ‘emotional’ and the ‘cognitive’ brain is not relevant to correctly interpret animal behavior as putatively emotional and cognitive regions influence each other via a complex web of connections that contribute to adaptive and maladaptive behavior.

## Materials and methods

### Animals and treatments

Two distinct experiments were performed, each dedicated to a specific dose of CYP. We first performed experiment 1 with a moderate dose of 20 mg/kg bw (the 2 experimental groups will be noted as CTL1 and CYP20). Experiment 2 was performed with 5 mg/kg bw CYP (the 2 experimental groups will be noted as CTL2 and CYP5). Seven-week-old female CBA/J mice were purchased from Janvier (Le Genest St Isle, France). All mice were bred and maintained on a 12-h light/dark cycle (lights on from 7:00 a.m. to 7:00 p.m.) with food and water ad libitum in a temperature controlled (21 ± 1°) room in our EOPS animal resource facility (UPS44, CNRS Orléans—France). After an acclimation period of 2 weeks, two female mice were paired with one male CBA/J mouse also from Janvier (Le Genest St Isle, France), in standard laboratory cages (42 cm × 28 cm × 18 cm) to mate. Pregnant mice were then isolated and divided into 2 experimental groups treated intranasally with CYP 20 mg/kg bw or vehicle solution (3% DMSO– 3% Tween 80 in NaCl 0.9%) in experiment 1 and with 5 mg/kg bw or vehicle solution (1% DMSO– 1% Tween 80 in NaCl 0.9%) in experiment 2. In each experiment, CYP was obtained from Sigma–Aldrich (PESTANAL^®^, analytical standard; 60:40 trans-cis). Intranasal exposure was performed by administration of 10 μL of solution for a 30 g mouse deposited at the entrance to the nostrils. Dams were treated three times a week, from gestational day (GD) 6–7 to postnatal day 15 (PND 15). Control animals received a comparable dose of vehicle solution. Offspring were weaned at 21 days of age and maintained in same-sex, litter-mate housed cages with ad libitum access to food and water. Experiment 1 involved 41 females: 20 CTL1 and 21 CYP20. Experiment 2 involved 27 females: 13 CTL2 and 14 CYP5. All aspects of animal care and experimentation were in accordance with the European Parliament and Council Directive (2010/63/EU). The Ethics Committee approved all animal care used for this study (Comité d’éthique en expérimentation animale Campus CNRS d’Orléans; Approval C45-234-6).

### General procedure

The general procedure was similar to that used in our previous studies [12, 13]. Mating was performed within 5–6 days. At the end of this period, males were removed and dams were weighed every 2 days. Females that gained 2g were considered pregnant and were isolated in standard cages with nesting material (pressed cotton). Intranasal treatment began the same day and lasted until PND 15. Each female received 10 treatments (4 during pregnancy and 6 during lactation) at a rate of 3 treatments a week (on Monday, Wednesday, and Friday; between 9:00 and 12:00 a.m.). To verify whether intranasal exposure to CYP induced behavioral changes that could account for abnormal maternal behavior in dams, clinical observations were performed before each treatment session, *i*.*e*. every 2 days. Several parameters were checked such as nesting abilities, hyperactivity in response to handling, pup retrieval and infanticide behavior. From these observations, a score of maternal distress was calculated for each dam as follow: for nesting abilities: 0 = shaped nest with all cotton used; 1 = cotton used but flat nest; 2 = no nest, no cotton used. For hyperactivity in response to handling: 0 = normal behavior; 1 = mild hyperactivity and 2 = important hyperactivity. For pup retrieval: 0 = short latency to retrieve pups after testing *i*.*e*. within 10–20 seconds and 1 = increased latency to retrieve pups after testing (more than 1 min). For infanticide behavior: the total number of dead pups was determined. During each session of clinical observation, all the dams were assessed as described above and each score was added to constitute a final score on the physical and mental wellbeing of pregnant and lactating dams. An elevated score indicated maternal distress.

One day after birth, all pups were weighed and then tested in a series of pre-weaning tests to check the effects of perinatal exposure to CYP on postnatal development. From PND 1 to 15, we investigated pups’ communication by monitoring ultrasonic vocalizations (USVs). Immediately after the 3-min period of USV collecting, we assessed early sensory and motor development by monitoring 7 parameters in all pups: the righting reflex (from PND 1 to 10), walking and 45° negative geotaxis (from PND 4 to 10), vertical climbing and bar grasping (from PND 8 to 14), and the day of eyelid opening (from PND 10 to 14) and acquisition of the acoustic startle reflex (from PND 8 to 14). Duration of this testing period was no more than 5 min per pup.

Data from each pup in all of the litters was collected and then averaged so the litter was used as the unit for statistical analyses. At the end of the exposure (PND 15), 4 CTL males and 4 CYP males (each from a different litter) were euthanized for whole brain transcriptomic analyses. Remaining siblings were allowed to grow until weaning. At PND 21, siblings were weaned. Only male offspring were reared by the litter and left undisturbed until the age of 3 months to conduct adult behavioral analyses. For adult testing in experiment 1, the groups’ composition was as follows: n = 20 (from 6 litters) for the CTL group and n = 21 (from 6 litters) for the CYP20 group. In experiment 2, the groups’ composition was as follow: n = 13 (from 4 litters) for the CTL group and n = 14 (from 5 litters) for the CYP5 group. Adult behavioral testing was performed on male offspring only as CYP has been shown to be a potent antiandrogenic chemical and a potent disruptor of reproductive functions in male mice [15, 16]. OECD 426 recommended including tests of a higher function in the developmental neurotoxicity evaluation of toxicants [11]. In agreement with this, we used ethological procedures such as context-dependent testing and repeated testing to collect more behavioral endpoints in a limited number of tests. By doing so, our approach adhered to the “Refinement” process of the 3R principles (Russell, 1959).

### Neurobehavioral assessment: Pre-weaning tests

#### Ultrasound vocalizations

Vocalizations were recorded from PND 1 to 15; each animal was tested daily during a 3-min period using the following experimental setup: a custom designed recording chamber made of transparent Plexiglas, 4 ultrasound detectors (UltraVox 4-channel system; Noldus Information Technology), and data acquisition software (UltraVox 2.0; Noldus Information Technology), which automatically monitored the occurrence of vocalizations within user-defined frequencies (in our case: 20, 40, 60, and 80 KHz). The number of USVs was recorded on a personal computer for offline analysis and storage. The computerized recording system was set to eliminate non-relevant sounds (background noise) and to ignore ultrasounds outside the defined frequency range. Ultrasonic calls were recorded for 3-min periods, between 9:00 a.m and 12:00 p.m. (noon), in an experimental room maintained at approximately 21°C. A single pup was removed from the litter and placed in a square container (5 cm × 5 cm; height, 2 cm) at the center of a sound-attenuating chamber, and USVs were assessed. All the pups were individually tested in turn. The container had been saturated with maternal odor to avoid environmental stress and had been cleaned between runs. After the 3-min recording session, each pup went through all the other pre-weaning tests. Each day we measured the number of calls emitted during the 3-min period for each pup. The litter was used as the unit for statistical analyses.

#### Postnatal sensory and motor development

The investigation of early postnatal sensory and motor development monitored 6 parameters:

- Negative geotaxis: The pup turned upwards when placed on a 45° angle slope with its head pointing down the incline (PND 4–10). Each day we recorded the percentage of pups that acquired the reflex.
- Righting reflex: When a pup is placed on its back on a flat, hard surface, it has to right itself on all four paws, with two successes in three trials getting a score of 1. A failure is recorded when the pup remains on its back for more than 10 s, and a score of 0 is given. Each day we recorded the percentage of successful pups. A pup was recorded as successful when it got two successes in three trials (from PND 1 to 10).
- Vertical progression: The pup is held against a vertical metallic grid (mesh: 6 mm wide). Climbing was scored when the pup passed through 5 grids (PND 8–14).
- Bar grasping: The forepaws are placed on a round wooden bar (7 mm diameter). The ability to hang for 10 s using the forepaws was scored (PND 8–14).
- Eyelid opening: Defined as any visible break in the membrane covering the eye. We examined pups from PND 10 to 14 and determined the day of eyelid opening (pups with at least one eyelid opened).
- Acoustic startle reflex: From PND 8 to 14, a small hand-held clicker generated a loud noise and the jerk behavior immediately following was scored. The day of reflex acquisition was noted.

Each pup was tested for all of these tests and their scores averaged per litter. The litter was used as the unit for statistical analyses.

#### Whole brain RNA extraction

At the end of the treatment (PND15), 4 males CTL1 vs 4 males CYP20 on the one hand and 4 males CTL2 vs 4 males CYP5 on the other hand (each from a different litter) were euthanized with CO_2_ and decapitated in order to extract whole brain RNAs. Extraction was performed using Trizol reagent (Invitrogen, Carlsbad, CA) following the manufacturer's instructions. Quantity and quality of the total RNA were controlled by Nanodrop spectrophotometer (Nanodrop, Wilmington, DE) and Agilent Bioanalyser (Agilent technologies, Palo Alto, CA) in accordance with manufacturer's instructions. In samples A260/A280 the absorbance ratio was greater than 1.8 and 28S/18S rRNA ratio greater than 1.5.

#### Affymetrix mice exon 1.0 ST array 1.0 and micro-arrays analyses

Gene expression was tested by the Affymetrix Mice Exon 1.0 ST Array (Affymetrix, Santa Clara, CA). A total of 250 ng of total RNA from each brain sample was labeled with reagents from Affymetrix according to manufacturers' instructions. Hybridization cocktails containing 5–5.5 μg of fragmented, end-labeled single-stranded cDNA were prepared and hybridized to GeneChip Mouse Exon 1.0 ST arrays. Arrays were washed, stained and scanned on the Affymetrix Fluidics Station and G7 Affymetrix high-resolution scanner (GCOS 1.3). Affymetrix Expression Console Software^™^ (version 1.0) was used to perform quality assessment. Raw signals were then transformed into “.CEL” files in GCOS software (Affymetrix, Santa Clara, CA). GeneChip CEL files (.CEL file contains probe cell intensity data) were analyzed using GeneSpring (Agilent Technologies). The log2 values were obtained and the microarray data was normalized using this algorithm RMA16 (Robust Multi-chip Average). Statistical data analyses were done on all samples for each group. Similarly, enrichment rankings were based on all samples per group. Data was filtered with GeneSpring analysis using a cutoff of at least 1.2× up or down (Fold change 1.2, FC1.2). Subsequently, one-way ANOVA was performed on a filtered list. The Benjamini–Hochberg false discovery rate (FDR) was set to 5%, and the P value was set to < 0.05, ANOVA). Quantitative PCR was used to validate the expression arrays, through the validation of 45 genes.

The Database for Annotation, Visualization, and Integrated Discovery (DAVID) was used to find gene ontology-enriched (GO) terms (Dennis et al., 2003; Huang et al., 2009). Furthermore, to determine significant enrichment of a class of genes, the list of the genes in each category was compared with the list of all open reading frames present in the complete dataset. The GO Term Finder evaluated the statistical significance of the association of a particular GO term with a group of genes in the list by calculating the P-value. That is, the probability, or chance, of seeing at least x number of genes out of the total n genes in the list annotated to a particular GO term, given the proportion of genes in the whole genome annotated to it. That is, the GO terms shared by the genes in the user’s list are compared with the background distribution of annotation. The particular GO term associated with the group of genes was more significant when the P-value was closer to zero. This indicated it was less likely that the observed annotation of the particular GO term to a group of genes occured by chance.

Functional classification of the modulated genes was performed on group terms with similar biological meaning due to sharing similar gene members assigned to a specific gene in the categories of biological process (BP), molecular function (MF) and cellular component (CC) of the GO database. Here, we reported on the GO categories that were significantly modulated and on a number of genes chosen according to different criteria: genes modulated by CYP5 and CYP20.

### Adult behavior

**Fig 1:**
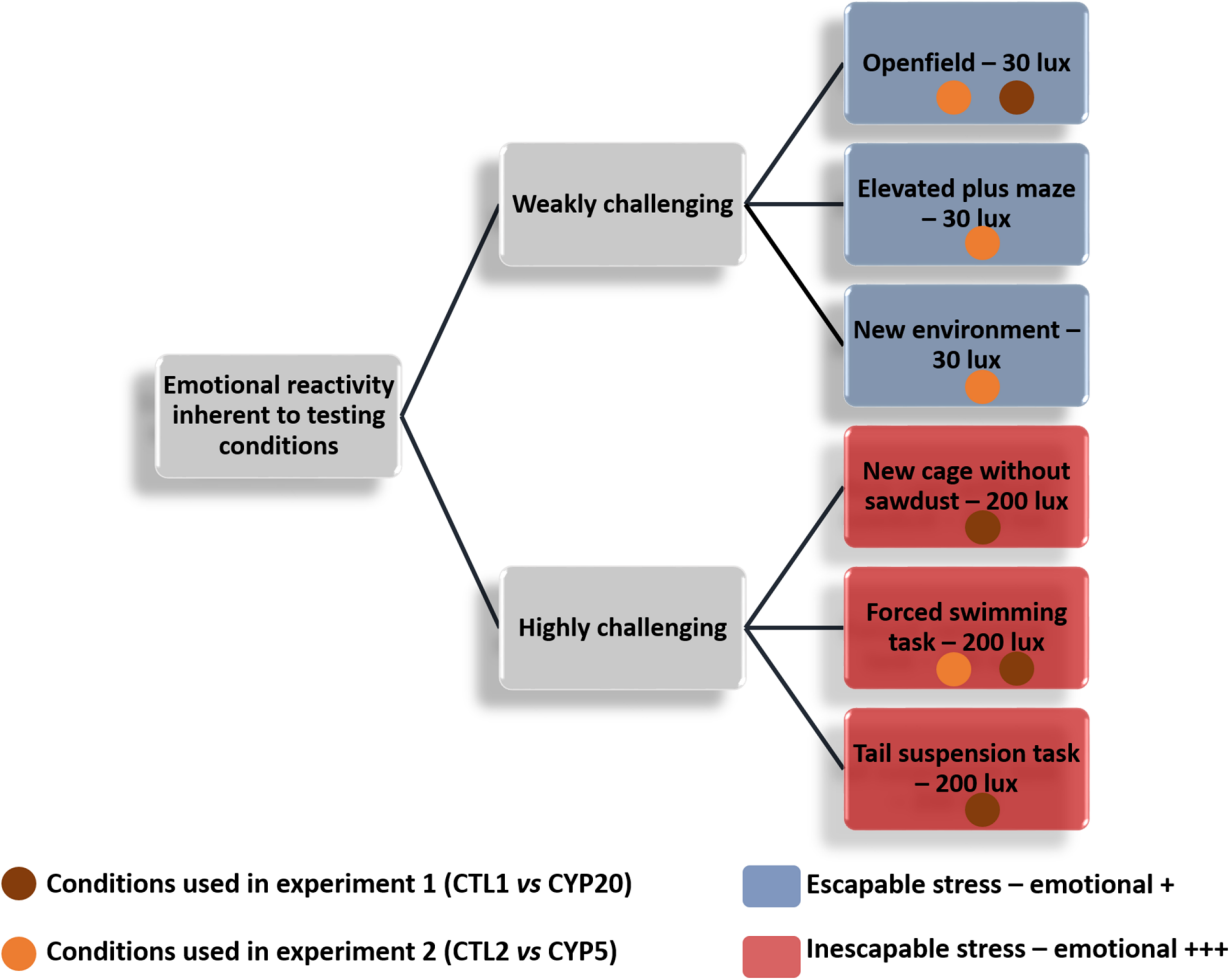
Behavioral tests used in the two experiments. Control and CYP-exposed offspring were exposed to both weakly and highly emotionally challenging environments. Conditions were defined as weakly challenging when the animals could escape from the stress generated by the apparatus and luminosity was low (no more than 30 lux). On the contrary, conditions were defined as highly challenging when the animals were not able to escape from the stress generated by the apparatus and luminosity was high (200 lux). The orange circles show the conditions for the CYP5-exposed offspring while the brown circles show the conditions for the CYP20-exposed offspring.

As previously stated, long-lasting consequences of perinatal exposure to CYP on the offspring’s behavior were assessed through finely tuned and ethologically relevant analyses. For instance, we routinely used minute-by-minute analysis to provide informative data on the way animals immediately react to their environment (first minutes in stressful testing conditions are more importantly emotionally charged) and also on the strategy they use to cope with subsequent unpleasant conditions (animals may adapt differently, later in the test, to stressful conditions, *i*.*e*. they may habituate differently). The level of emotional response inherent to testing conditions was also varied with the aim of refining behavioral analyses through detection of differences that could be context-specific. Consistent with this, we confronted CTL and CYP offspring to both weakly and highly emotionally challenging environments. Conditions were defined as weakly challenging when the animals could escape from the stress generated by the apparatus and luminosity was low (no more than 30 lux). On the contrary, conditions were defined as highly challenging when the animals were not able to escape from the stress generated by the apparatus and luminosity was high (200 lux). By using this procedure, we could make the testing environment more or less challenging and consequently collect more accurate data on behavioral changes induced by CYP exposure. In addition, varying stress controllability among the tests could provide significant information on CYP-induced defects as it is known that coping strategies (and their underlying neural mechanisms) in response to escapable and inescapable stressors are quite different [17, 18]. These procedures were an integral part of the 3Rs refinement process of our method [19]. All the conditions used in experiments 1 and 2 are indicated in Fig 1.

#### Weakly emotionally challenging conditions

Openfield: The openfield was used to assess the offspring’s ability to cope with escapable stressful situations. The set-up consisted of a dimly illuminated (indirect light; 30 lux in the center) squared arena (49 cm side) made of grey Plexiglas. Mice were individually placed in a corner of the openfield and allowed to explore the entire apparatus for 5 min. Automatic monitoring recorded the total distance travelled in the arena and the time spent in 3 distinct zones − the center, the intermediary and the periphery. Mean speed in each zone was also recorded. Analyses were performed using the EthoVision video-tracking system (Noldus, The Netherlands). The apparatus was cleaned between subjects using a 50% ethanol/water solution.

Elevated plus maze: The Elevated plus maze (EPM) is known to detect anxiety-like behavior in rodents [20, 21]. In this work, we used this task to assess offspring’s ability to cope with escapable, stressful situations. The apparatus had a central hub (5 cm × 5 cm), and two pairs of diametrically opposed arms, one pair open with no sides (27 cm × 5 cm) and the other pair fully enclosed (27 cm × 5 cm × 15 cm). The maze was made of black Plexiglas, elevated to a height of 60 cm and the open arms were lit by bulbs providing 35 lux at the far end of each. The 5 min test began by placing an individual mouse at the end of an enclosed arm. A video camera mounted above the maze recorded the trials for later analysis. The apparatus was cleaned between runs with a 50% ethyl alcohol solution. Total distance traveled in the apparatus was used as an index of stress-induced locomotor activity. Time spent in the anxiogenic open arms was used as a conventional measure of spatial avoidance. Time spent in stretched attend postures was also monitored as an index of risk assessment behavior. Time spent in the central hub area and the closed arms were also measured. All the analyses were performed using the EthoVision^®^ video-tracking system (Noldus Inc., The Netherlands). Twenty-four hours after the first trial, each animal was re-tested in the same apparatus. It is known that the EPM re-test effect can show a biologically adaptive form of learning/memory where the experience of a novel environment will swiftly lead to less time spent in the unsafe open arms [22].

Exposure to a new environment: The first trial of the three-chambered sociability test (habituation trial) was used to assess the offspring’s emotional cognition in response to a novel environment (see below for a full description of the apparatus). The arena was weakly illuminated (30 lux—indirect light) in order to be slightly challenging. Total distance traveled in the apparatus was used as an index of stress-induced locomotor activity. Measurements were performed using the EthoVision^®^ video-tracking system (Noldus Inc., The Netherlands). Each mouse was allowed to explore the arena for 5 min.

#### Highly emotionally challenging conditions

Novel cage test with no sawdust: Mice were run individually in a novel standard cage (42 cm × 28 cm × 18 cm) containing no sawdust, a testing condition recognized to be emotionally challenging [23]. The apparatus was highly illuminated (direct light; 200 lux) in order to increase the intensity of stress during testing. Such a set-up was used as highly emotional stressor from which mice could not escape *i*.*e*. inescapable stressor. The animals were free to explore their environment for 10 min. The offspring’s ambulatory exploration was monitored by the EthoVision video-tracking system (Noldus, The Netherlands) as it is known to be a reliable marker of emotional state changes in mice [24].

Forced swimming task: This task was used as a highly stressful condition from which the mouse could not escape. It was performed according to standard published procedures with minor modifications [25, 26]. Mice were placed in a glass cylinder (12 cm diameter, 25 cm tall) filled to a depth of 10 cm with water (22+/-1°C). A 6 min. test session was conducted and videotaped for a later analysis using the EthoVision video-tracking system (Noldus, The Netherlands). The time during which mice were immobile was measured. A mouse was considered immobile when it floated passively performing only slight movements of its limbs and tail without any gross head movements that could be judged as searching. “Immobility” included slow drifting on the water surface without any initiation of swimming. During the 6 min task, immobility was dynamically analyzed in order to collect data related to early emotional reaction and later behavioral habituation/adaptability [27]. In experiment 1, all mice were re-tested in this task to evaluate their ability to habituate to this inescapable condition.

Tail suspension task: As in the forced swim task, the tail suspension task was used as a highly emotional inescapable stressor. In this task, each mouse was suspended by its tail on a hook, 15 cm from the floor, using adhesive tape (approximately 0.5 cm from the base of the tail). A 10 min test session was conducted and videotaped for a later analysis using the EthoVision^®^ video-tracking system. The time during which mice were immobile was measured. A mouse was considered immobile when it stopped struggling and hung without any movement.

Social skills—three-chambered sociability test: Social dimension of the offspring’s behavior was addressed only during experiment 2 (CTL2 vs CYP5). Mice were run individually in a three-chambered arena made of clear polycarbonate. Retractable doorways built into the two dividing walls controlled access to the side chambers. Each of the two outside chambers had an inverted empty wire cup, one side housing a male C57Bl6/J stimulus mouse age-matched to the test mouse, and the other side with a plastic object (plastic mouse) or a novel stimulus mouse. The test consisted of 3 consecutive 5-min trials. The first trial (habituation trial) began with a 5-min habituation session with the mouse free to explore the entire arena. In the second trial (sociability trial), the subject was briefly confined to the center chamber while the plastic object was placed in the cup on one side and an adult male C57Bl6/J mouse was placed in the cup on the other side. In the last trial, the plastic object was substituted with a novel adult male C57Bl6/J mouse. The object/novel mouse sides were alternated left and right between subjects. Once the stimuli were in position, the two side doors were simultaneously raised so the subject could access all three chambers for 5 min. Automatic monitoring recorded and scored the time spent in contact with each wire cup using the EthoVision video-tracking system (Noldus, The Netherlands). The apparatus was cleaned between subjects using a 50% ethanol/water solution.

Social skills—direct interaction with peers: Social interaction was tested in a novel cage (24 cm × 11 cm × 12 cm) with clean sawdust. Each mouse was paired with either an unfamiliar NMRI female or an unfamiliar C57Bl6/J male mouse according to the experiment. The opponent mice were age-matched to the test mouse. The test mouse was isolated for 30 min before the unfamiliar conspecific was placed in the cage. Social interaction between the two mice was recorded using a digital camera mounted above the cage at ceiling level. The paired mice had never interacted before. The videos were analyzed by a trained observer in a blinded condition. Social investigation is a complex behavior and was assessed as such by using a microstructural analysis *i*.*e*. distinguishing body parts of interest. In this way, we monitored time spent sniffing the head, body and anogenital zones of the conspecific by the test mouse. We also measured time spent expressing non-social behaviors such as auto-grooming and rearing exploration of the cage. Each of these parameters was investigated in a time-dependent manner through a minute-by-minute analysis.

### Statistical analysis

Neurodevelopmental endpoints: Litter was used as the unit of analysis for USVs, all early sensorimotor endpoints and body weight data. The number of USVs and body weight were analyzed using 2-way ANOVA with repeated measures. A probability value of less than 0.05 was considered to be statistically significant. When necessary, *post hoc* analyses were done using Dunn-Sidak comparisons to minimize the risk of a type-1 error resulting from multiple comparisons. Differences on early developmental endpoints (comparisons between 2 proportions) were analyzed using Chi-squared statistics.

Adult behavior: In order to reduce variability inherent to adult behavioral data, we used modern robust statistical methods to maximize accuracy and power of our study [28]. In this way, dependent variables were 15% winsorized and then parametric statistical procedures were used. Independent two-group comparisons were analyzed using paired / unpaired t-tests. Minute-by-minute analyses were analyzed using 2-way repeated ANOVA. A probability value of less than 0.05 was considered to be statistically significant. When necessary, *post hoc* analyses were done using Dunn-Sidak comparisons.

Transcriptomic data: For statistical analysis of transcriptomic data, see the “Affymetrix mice exon 1.0 ST array 1.0 and micro-arrays analyses” section. Graphpad prism was used to perform the statistical analyses.

## Results

### Maternal effects

**Fig 2:**
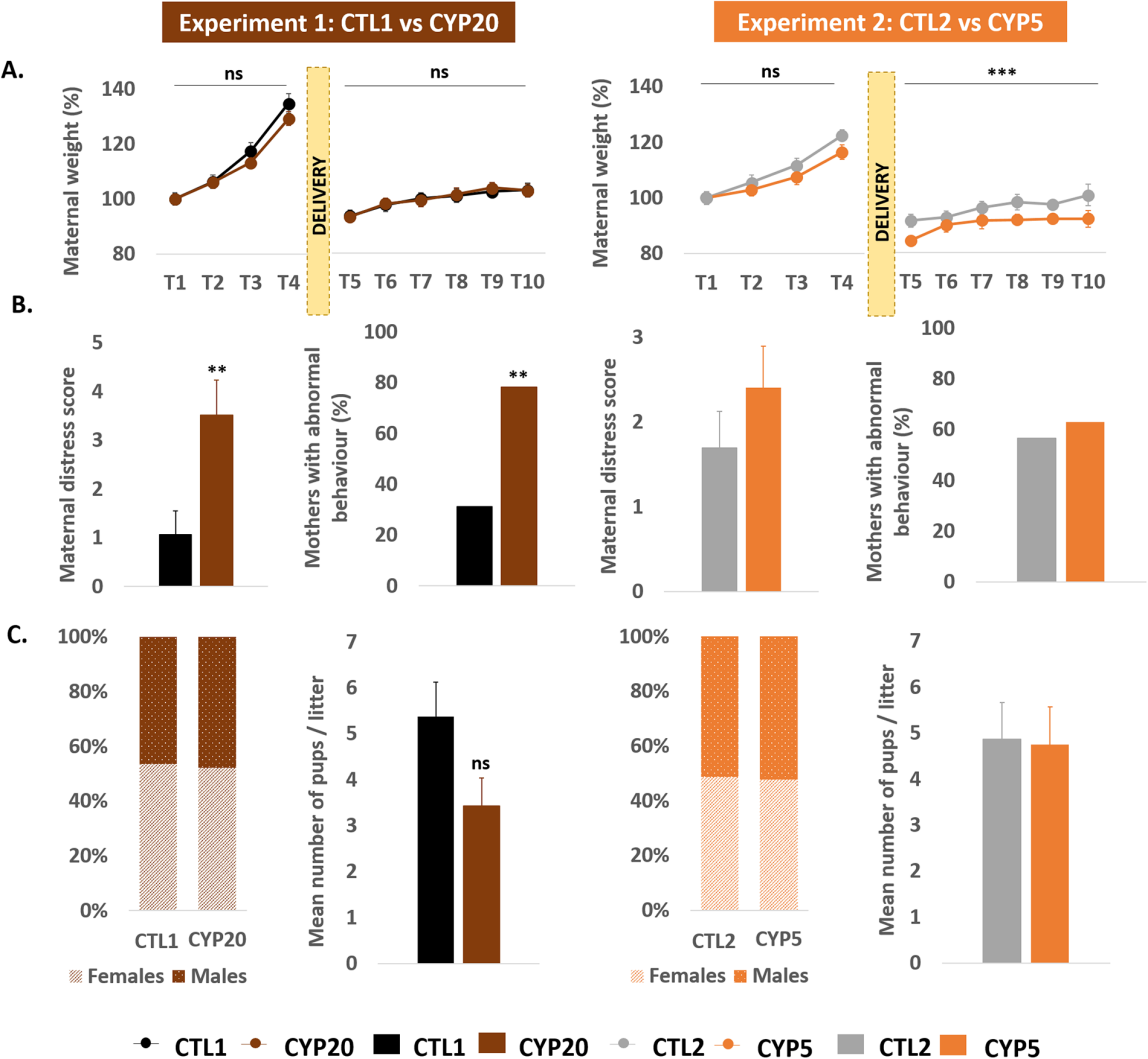
General toxicity induced by perinatal exposure to cypermethrin. **A**: Cypermethrin did not modify maternal weight gain during pre and postnatal exposure. Maternal weight gain was evaluated every two days, during the pre and postnatal period. **B**: Each treatment session was associated with a clinical and behavioral assessment of the mothers (see Materials and methods for details). **C**: On the day of delivery, sex ratio within each group and the number of pups by litter were determined. Data are mean +/- sem; Experiment 1: n = 20-21/group; Experiment 2: n = 13-14/group. ** p < 0.01 and *** p < 0.001 compared to CTL.

In order to assess whether perinatal exposure to CYP might directly alter maternal physiological and/or behavioral parameters, dams were weighed and their maternal behavior monitored all along the treatment period. Data revealed no significant adverse effects of CYP20 on body weight gain whereas CYP5 females displayed reduced postnatal body weight gain compared to CTLs (main effects of “Time” [*F*_5,222_ = 5.03; p = 0.0002] and “Group” [*F*_1,222_ = 18.9; p < 0.0001]) (Fig 2A). CYP20 significantly increased maternal distress during the early postnatal period (p = 0.006) while no effect was observed in CYP5-exposed females (Fig 2B). Indeed, almost 80% of CYP20 females had a maternal distress score higher than 0 against only 31% in CTL females (p = 0.0012). In spite of these alterations, neither the mean number of pups by litter nor the male / female ratio were altered by CYP exposure (Fig 2C).

### Brain maturation during early postnatal period

**Fig 3:**
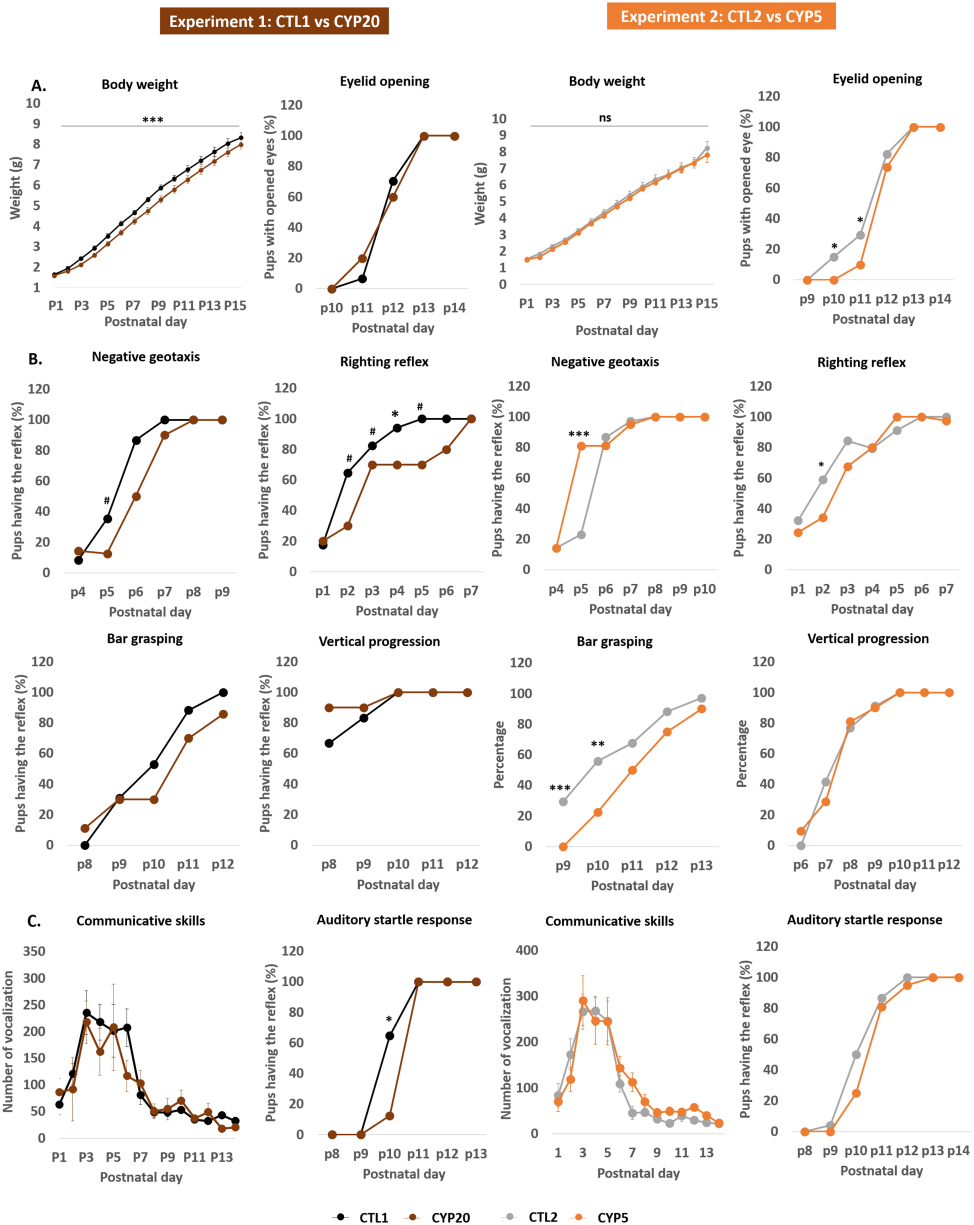
Perinatal exposure to cypermethrin disturbed early postnatal development in male offspring. **A**: Somatic growth and maturation endpoints revealed no significant effect of CYP. Body weight data are mean +/- sem; n = 20–21 /group in experiment 1 and n = 13–14 in experiment 2. Eyelid opening data are percent of pups with at least one eye opened. **B**: Neuromotor and reflex development investigate the state of motor development and its coordination, and its ability to maintain balance. CYP negatively impacted these parameters. Percentage of pups with the corresponding reflex. **C**: Communicative and auditory sensory functions in response to perinatal exposure to CYP. Communicative skills data are expressed in mean +/- sem; n = 20–21 /group in experiment 1 and n = 13–14 in experiment 2. Data related to auditory startle response are expressed as percent of pups having the reflex. *** p < 0.001, ** p < 0.01, *p < 0.05 and # p < 0.09.

Developmental endpoints are reported by categories as previously recommended [29]. Each category was informative on the various aspects of the offspring’s neurodevelopment. Somatic growth and maturation of the offspring were assessed through body weight gain from PND1 to PND15 and eyelid opening as recommended. Related data indicated that CYP5 did not alter early life body weight gain while CYP20-exposed offspring were lighter than their control counterparts (main effects of “Group” [F_1,355_ = 42. 8; p < 0.0001], “Day” [F_14,355_ = 634.8; p < 0.0001]; no “Group x Day” interaction) (Fig 3A). CYP5 exposed offspring displayed delayed eyelid opening compared to CTLs while CYP20 offspring were not different from their CTL counterparts (Fig 3A). Neuromotor development was also investigated through the appearance of various reflexes such as negative geotaxis, righting reflex, bar grasping and vertical progression. Results showed that the 2 doses of CYP had a negative impact on early neuromotor development as CYP20 significantly delayed the appearance of negative geotaxis and righting reflex and CYP5 significantly impacted negative geotaxis, righting reflex and bar grasping (Fig 3B). Then, sensory functions and communicative skills were addressed through the measurement of ultrasonic calls and auditory startle responses. Two way repeated ANOVA revealed that neither one of the two doses exerted negative effects on the offspring’s ability to emit ultrasonic calls in response to maternal separation. However, the auditory startle reflex was acquired more slowly in CYP20 offspring while no effect was observed in CYP5 offspring (Fig 3C).

### Morphological changes at the end of the treatment

Morphological effects of CYP exposure were investigated at the end of the treatment period *i*.*e*. PND15, through measurements of brain and testis weight (Table 1). Data indicated that none of the 2 doses induced changes to brain or testis weight.

**Table 1:** Morphological effects observed at the end of the treatment period (PND15).

	Absolute brain weight (mg)				Absolute testis weight (mg)				Relative brain weight				Relative testis weight			
	CTL2	CYP5	CTL1	CYP20	CTL2	CYP5	CTL1	CYP20	CTL1	CYP5	CTL2	CYP20	CTL2	CYP5	CTL1	CYP20
mean	396.6	394.0	393.3	398.5	10.3	9.8	9.6	9.2	8.1	8.9	8.1	8.0	0.21	0.22	0.2	0.18
sem	4.1	4.3	3.7	5.1	0.8	0.3	0.4	0.8	0.2	0.3	0.3	0.2	0.01	0.01	0.005	0.02

### Whole brain transcriptomic changes at the end of the treatment

DNA microarray analysis revealed 83 dysregulated genes in CYP20 groups (Data in S1 Table) and 54 significantly dysregulated gene transcripts in CYP5 (Data in S2 Table) compared to CTL condition (>FC 1.2).

#### In CYP20 condition (Table 2)

**Table 2:** Functional annotation charts for dysregulated genes in the brain of CYP20 and CYP5-exposed offspring.

**Biological Process Chart—CYP20**			
**Term**	**PValue**	**Genes**	**Benjamini**
GO:0006412~translation	1.11E-04	RPS18, MRPL14, RPL13, RPL35, FAU, RPL37, RPS27A, GM10269, RPL29	2.30E-02
GO:0007608~sensory perception of smell	7.71E-04	OLFR539, OLFR420, OLFR599, OLFR447, OLFR150, OLFR1338, OLFR1009, OLFR1155, OLFR666, OLFR32, OLFR741, OLFR10, OLFR919	7.78E-02
**Molecular Function Chart—CYP20**			
**Term**	**PValue**	**Genes**	**Benjamini**
GO:0003735~structural constituent of ribosome	1.00E-06	RPS18, GM3550, MRPL14, RPL13, RPL35, FAU, RPL37, RPS27A, GM10269, RPL29	1.15E-04
GO:0004984~olfactory receptor activity	1.52E-03	OLFR539, OLFR420, OLFR599, OLFR447, OLFR150, OLFR1338, OLFR1009, OLFR1155, OLFR666, OLFR32, OLFR741, OLFR10, OLFR919	8.39E-02
GO:0004930~G-protein coupled receptor activity	2.66E-03	OLFR539, OLFR420, OLFR599, TAS2R102, OLFR447, OLFR150, OLFR1009, OLFR1338, OLFR1155, OLFR666, OLFR32, VMN2R7, OLFR741, LTB4R2, OLFR919, OLFR10	9.71E-02
**Cellular Compound Chart—CYP20**			
**Term**	**PValue**	**Genes**	**Benjamini**
GO:0022625~cytosolic large ribosomal subunit	1.60E-05	GM3550, RPL13, RPL35, RPL37, GM10269, RPL29	1.19E-03
GO:0022627~cytosolic small ribosomal subunit	4.49E-05	HBA-A1, HBA-A2, RPS18, FAU, RPS27A	1.66E-03
GO:0005840~ribosome	5.03E-04	RPS18, MRPL14, RPL13, FAU, RPS27A, RPL29	1.23E-02
GO:0015935~small ribosomal subunit	5.28E-03	RPS18, FAU, RPS27A	9.33E-02
**Biological Process Chart—CYP5**			
**Term**	**PValue**	**Genes**	**Benjamini**
GO:0045653~negative regulation of megakaryocyte differentiation	9.88E-08	HIST1H4M, HIST1H4A, HIST1H4B, HIST1H4F, HIST1H4C	3.28E-05
GO:0006336~DNA replication-independent nucleosome assembly	3.39E-07	HIST1H4M, HIST1H4A, HIST1H4B, HIST1H4F, HIST1H4C	5.63E-05
GO:0032776~DNA methylation on cytosine	1.29E-06	HIST1H4M, HIST1H4A, HIST1H4B, HIST1H4F, HIST1H4C	1.42E-04
GO:0045815~positive regulation of gene expression, epigenetic	1.45E-06	HIST1H4M, HIST1H4A, HIST1H4B, HIST1H4F, HIST1H4C	1.21E-04
GO:0006335~DNA replication-dependent nucleosome assembly	1.45E-06	HIST1H4M, HIST1H4A, HIST1H4B, HIST1H4F, HIST1H4C	1.21E-04
GO:0006352~DNA-templated transcription, initiation	2.30E-06	HIST1H4M, HIST1H4A, HIST1H4B, HIST1H4F, HIST1H4C	1.53E-04
GO:0051290~protein heterotetramerization	5.47E-06	HIST1H4M, HIST1H4A, HIST1H4B, HIST1H4F, HIST1H4C	3.02E-04
GO:0006334~nucleosome assembly	6.02E-06	HIST1H4M, HIST1H4A, HIST1H4B, CENPA, HIST1H4F, HIST1H4C	2.85E-04
GO:0000183~chromatin silencing at rDNA	1.11E-05	HIST1H4M, HIST1H4A, HIST1H4B, HIST1H4F, HIST1H4C	4.59E-04
**Molecular Function Chart—CYP5**			
**Term**	**PValue**	**Genes**	**Benjamini**
GO:0042393~histone binding	2.19E-04	HIST1H4M, HIST1H4A, HIST1H4B, HIST1H4F, HIST1H4C	1.97E-02
GO:0044822~poly(A) RNA binding	1.27E-03	ZFP36L2, HIST1H4M, EIF4E, RPS16, HIST1H4A, HIST1H4B, RPL15, HIST1H4F, HIST1H4C, RPS27A	5.60E-02
GO:0005179~hormone activity	2.51E-03	HAMP2, HAMP, PMCH, APLN	7.34E-02
GO:0019904~protein domain specific binding	5.18E-03	HIST1H4M, HIST1H4A, HIST1H4B, HIST1H4F, HIST1H4C	9.01E-02
**Cellular Compound Chart—CYP5**			
**Term**	**PValue**	**Genes**	**Benjamini**
GO:0031012~extracellular matrix	1.94E-07	HIST1H4M, RPS16, HIST1H4A, CD93, HIST1H4B, RPL9, F3, HIST1H4F, HIST1H4C	1.69E-05
GO:0000786~nucleosome	4.00E-06	HIST1H4M, HIST1H4A, HIST1H4B, CENPA, HIST1H4F, HIST1H4C	1.74E-04
GO:0000228~nuclear chromosome	5.30E-06	HIST1H4M, HIST1H4A, HIST1H4B, HIST1H4F, HIST1H4C	1.54E-04
GO:0000784~nuclear chromosome, telomeric region	1.67E-04	HIST1H4M, HIST1H4A, HIST1H4B, HIST1H4F, HIST1H4C	3.62E-03
GO:0070062~extracellular exosome	1.25E-03	HIST1H4M, FKBP5, IFITM3, RPL15, CLDN5, PPT1, DBI, EIF4E, RPS16, HIST1H4A, HIST1H4B, F3, HIST1H4F, HIST1H4C, RPS27A	2.16E-02

The functional annotation chart showed 2 GO terms mainly enriched in the Biological Process (BP terms), 3 GO terms in Molecular Function (MF terms) and 4 GO terms in Cellular Compound (CC terms). Across all these GOs enriched for CYP20, two main axes were highlighted: 1) translation process and 2) olfactory perception. Translation process was characterized by the BP terms "translation" (GO: 0006412) enriched with the *RPS18*, *MRPL14*, *RPL13*, *RPL35*, *FAU*, *RPL37*, *RPS27A*, *GM10269* and *RPL29* genes. The same genes were classified in MF GOs "structural constituent of ribosome" (GO: 0003735) and CC GOs "cytosolic large ribosomal subunit" (GO: 0022625), "cytosolic small ribosomal subunit" (GO: 0022627), "small ribosomal subunit" (GO: 0015935) and "ribosome" (GO: 0005840). Olfactory perception was enriched with BP GOs "sensory perception of smell" (GO: 0007608) associated with MF GOs "olfactory receptor activity" and "G-protein coupled receptor activity" (GO: 0004984 and GO: 0004930, respectively). All these terms were related to the olfactory receptors genes *OLFR539*, *OLFR420*, *OLFR599*, *OLFR447*, *OLFR150*, *OLFR1338*, *OLFR1009*, *OLFR1155*, *OLFR666*, *OLFR32*, *OLFR741*, *OLFR10*, *and OLFR919*.

#### In CYP5 condition (Table 2)

Functional annotation chart showed 9 GO terms mainly enriched in the Biological Process (BP terms), 5 GO terms in Molecular Function (MF terms) and 4 GO terms in Cellular Compound (CC terms). BP terms were enriched with genes involved in the overall DNA regulation GOs such as "DNA replication-independent nucleosome assembly" (GO: 0006336), "DNA methylation on cytosine" (GO: 0032776), "nucleosome assembly" (GO: 0006334), "DNA replication-dependent nucleosome assembly" (GO: 0006335), or "positive regulation of gene expression epigenetic" (GO: 0045815). Interestingly, MF terms and CC terms were in accordance with these findings since MF GOs were focused on histone binding (GO: 0042393) or protein domain specific binding (GO: 0019904), and CC terms were related to nuclear chromosome (GO: 0000228, GO: 0000784) or nucleosome (GO: 0000786). Among these GOs, we mostly found gene of the histone cluster family (*HIST1H4A*, *HIST1H4B*, *HIST1H4C*, *HIST1H4M*, *HIST1H4F*), or chromatin remodeling factors (*CENPA*). We also observed that "hormone activity" MF GOs (GO: 0005179) was associated with *HAMP2*, *HAMP*, *PMCH* and *APLN* genes.

#### Among all transcripts dysregulated in CYP5 and CYP20 groups

The Venn diagram showed 8 genes commonly dysregulated in both CYP5 and CYP20 (Table 3). Cluster analysis showed similar profiles of gene expression for both groups. Indeed, 4 genes (*Olfr1155*, *Olfr420*, *Rps27a*, *Cox7c*) were down-regulated in both groups, whereas 3 genes (*Hamp׀Hamp2*, *Timm8q1*, *Olfr666*) were upregulated. In addition, the last gene (*Mertk*) displayed a different expression profile in CYP5 (Down-regulated) and CYP20 (Up-regulated). One transcript (*Hamp׀Hamp2)* was related to BP GOs "hormone activity" (GO: 0005179) and "iron transport" (GO: 0097690); 3 transcripts (*Olfr1155*, *Olfr420*, *Olfr666*) were related to BP "olfactory receptor activity" (GO: 0004984) and 2 transcripts (*Cox7c*, *Timm8q1*) were related to BP "mitochondrion electron transport" (GO: 0006123). Interestingly, this last observation on mitochondria was reinforced by the enrichment of mitochondrial GOs in CC terms as shown by GO: 0004739 "Mitochondrion" and GO: 0005751 "mitochondrial respiratory chain complex IV".

**Table 3:** Genes commonly dysregulated in the brain of CYP20 and CYP5-exposed offspring. Fold change ≥ 1.2.

Transcripts Cluster Id	FC ([Ctrl1] vs [Cyp20])	Log FC ([Ctrl1] vs [Cyp20])	Regulation ([Ctrl1] vs [Cyp20])	FC ([Ctrl2] vs [Cyp5])	Log FC ([Ctrl2] vs [Cyp5])	Regulation ([Ctrl2] vs [Cyp5])	Gene Description	Gene Symbol
6888531	-1.208	-0.272	**down**	-1.475	-0.561	**down**	olfactory receptor 1155	Olfr1155
6949693	-1.322	-0.402	**down**	-1.250	-0.322	**down**	ribosomal protein S27A	Rps27a
6789754	-1.322	-0.403	**down**	-1.297	-0.375	**down**	cytochrome c oxidase, subunit VIIc | cytochrome c oxidase, subunit VIIc pseudogene	Cox7c|Gm10012
6755306	-1.465	-0.551	**down**	-1.638	-0.712	**down**	olfactory receptor 420	Olfr420
6881087	1.208	0.273	**up**	-1.249	-0.321	**down**	c-mer proto-oncogene tyrosine kinase	Mertk
6966327	1.227	0.295	**up**	1.205	0.269	**up**	hepcidin antimicrobial peptide | hepcidin antimicrobial peptide 2	Hamp|Hamp2
6765982	1.286	0.363	**up**	1.224	0.292	**up**	translocase of inner mitochondrial membrane 8 homolog a1 (yeast)	Timm8a1
6970084	1.362	0.446	**up**	1.273	0.349	**up**	olfactory receptor 666	Olfr666

### Short-term emotional cognition

#### Weakly challenging conditions

**Fig 4:**
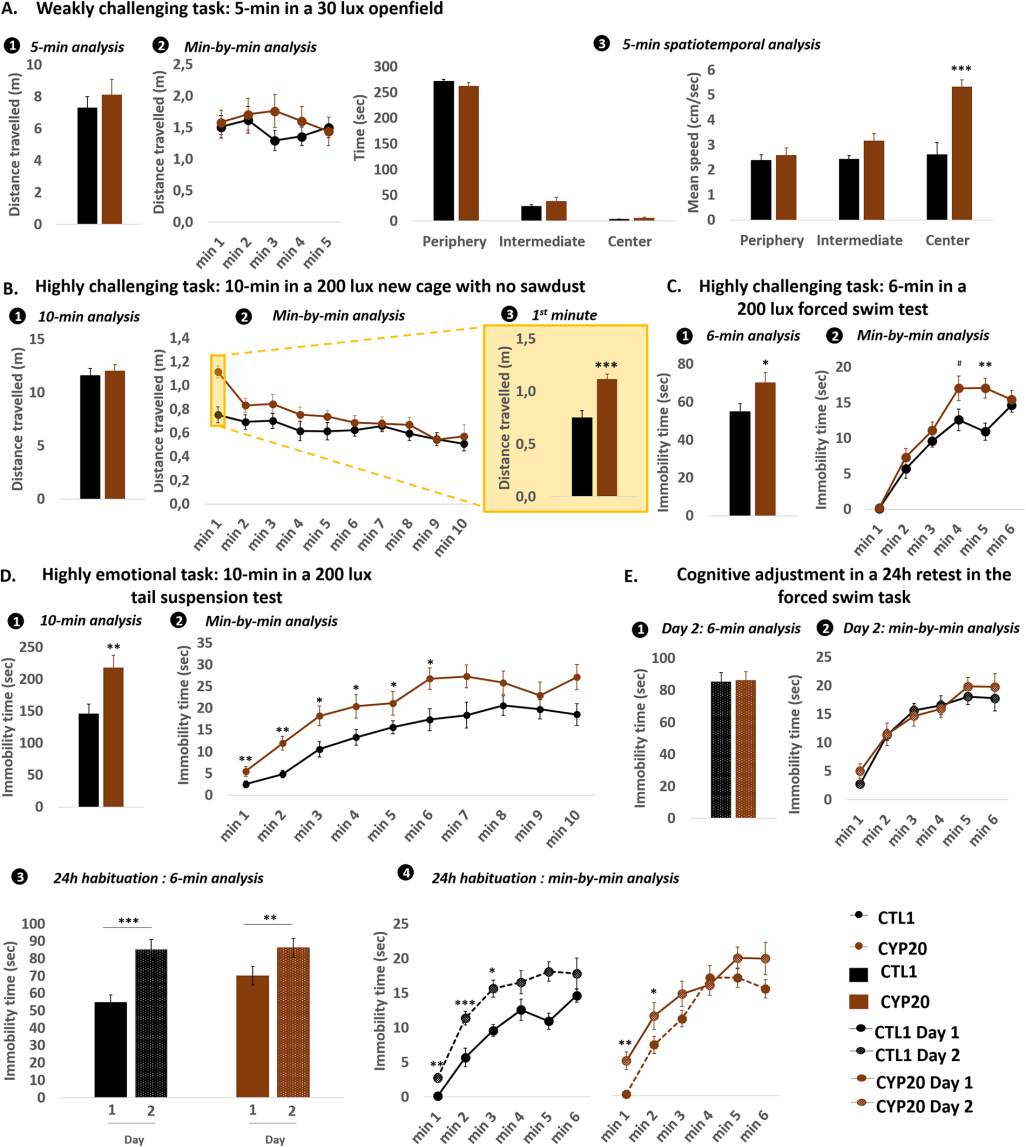
Adult CYP20-exposed offspring displayed maladaptive behavior in response to highly stressful conditions. **A**: The openfield was used as a weakly challenging task. Total distance travelled in the apparatus (1–2), time spent and the mean speed in different parts of the openfield were used to assess emotional reactivity (3). **B**: Emotional reactivity in response to highly challenging environments was evaluated by confronting mice to a novel cage with no sawdust. Total distance travelled was monitored over the total duration of the test (1). Data were also analyzed on a minute-by minute basis (2–3). **C-D**: Emotional reactivity in response to highly challenging environments was evaluated in the forced swimming task and the tail suspension task. In each condition, total time spent immobile was scored over the total duration of the test (1) and on a minute-by-minute basis (2). **E**: Habituation to a second exposure to stressful environments was assessed by confronting mice to the forced swimming task 24h later the first trial. Performance on day 2 is depicted on (1) and (2), and habituation between day 1 and day 2 is depicted on (3) and (4). Data are mean +/- sem; n = 20–21 /group. *** p < 0.001, ** p < 0.01, *p < 0.05 and # p < 0.09.

**Fig 5:**
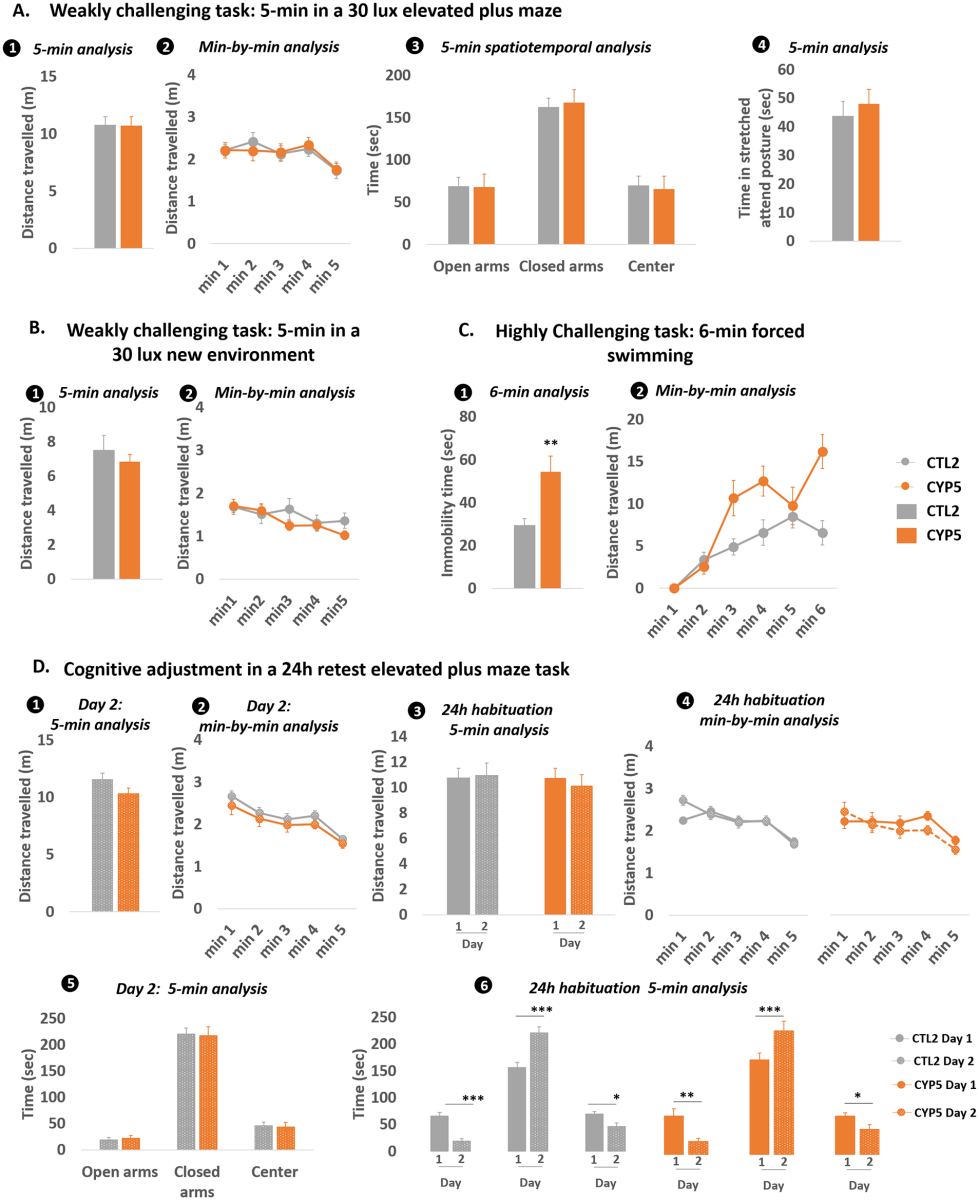
Adult CYP5-exposed offspring displayed maladaptive behavior in response to highly stressful conditions. **A**: The elevated plus maze (EPM) was used as a weakly challenging task. Total distance travelled in the apparatus (1–2), time spent and mean speed in different parts of the EPM were used to assess emotional reactivity (3). Time spent in stretched attend postures was also measured to collect data on risk assessment (4). **B**: Emotional reactivity in response to weakly challenging environments was also evaluated by confronting mice to a novel slightly lighted environment during 5 min: total distance travelled was monitored over the total duration of the test (1). Data were also analyzed on a minute-by minute basis (2). **C**: Emotional reactivity in response to highly challenging environments was evaluated in the forced swimming task by measuring total time spent immobile over the total duration of the test (1) and on a minute-by-minute basis (2). **D**: Habituation to a second exposure to stressful environments was assessed by confronting mice to the EPM 24h later the first trial. Distance travelled on day 2 is depicted on (1) and (2), and habituation between day 1 and day 2 is depicted on (3) and (4). (5) and (6) show how offspring habituate between day 1 and day 2 in terms of spatial exploration of the apparatus. Data are mean +/- sem; n = 13–14 / group. *p < 0.05, ** p < 0.01, *** p < 0.001.

As stated before, we used ethological tools to obtain finely-tuned data. We considered the ecological meaning of experimental conditions to which offspring were subjected. By doing so, we observed in our 2 experiments that when confronted to weakly challenging conditions, both CYP20 and CYP5-exposed offspring displayed normal ambulatory exploration (Figs 4A.1 and 5A.1). Minute-by-minute analysis did not reveal any difference between CYP and CTL offspring (Figs 4A.2 and 5A.2). The spatial analysis revealed that CYP5-exposed offspring did not differ from their CTL counterparts in exploring anxiogenic areas of the elevated plus maze (Fig 5A.3) while CYP20-exposed offspring displayed increased speed when visiting the center of the openfield (Fig 4A.3). Stretched attend postures, measured in the elevated plus maze, indicated that CYP5 did not affect risk assessment behaviors (Fig 5A.4). CYP5 offspring were also subjected to one more weakly challenging task and again, no difference from the CTLs could be observed (Fig 5B.1 and 5B.2).

#### Highly challenging conditions

When confronted during 10 min with a highly illuminated novel cage with no sawdust (an inescapable and more emotionally charged condition), ambulatory exploration of CYP20 offspring was similar to that of CTL offspring (Fig 4B.1). However, minute-by-minute analysis revealed that CYP20 offspring were hyperactive during the first minute of the task compared to CTLs (p = 0.0002) (Fig 4B.2 and 4B.3). By increasing stress intensity inherent to behavioral tasks, through the forced swim task and the tail suspension task, CYP20 mice also displayed increased immobility time compared to CTLs (p = 0.032 and p = 0.0062 respectively) (Fig 4C.1 and 4D.1). In the forced swim task, minute-by-minute analysis revealed that this phenomenon occurred mainly at the end of the test (main effects of “Group” [F_1,39_ = 5.62; p = 0.023], “Time” [F_5,195_ = 65.26; p < 0.0001] and “Time x Group” interaction [F_5,195_ = 2.56; p = 0.028]) while in the tail suspension task, the defect was observed earlier, from the second minute of test (Main effects of “Group” [F_1,39_ = 7.86; p = 0.0078], and “Time” [F_9,351_ = 32.18; p < 0.0001]) (Fig 4C.2 and 4D.2). In our second experiment, CYP5 offspring and their CTL conspecifics were also confronted with highly emotionally challenging tasks. Our results indicated that, similarly to CYP20 offspring, CYP5 offspring displayed maladaptive behavioral responses in the forced swim task, spending more time immobile (p = 0.0051) (Fig 5C.1). Consistent with our first experiment, minute-by-minute analysis revealed that CYP5-induced increase in immobility was observed from the second minute and lasted until the end of the test (main effects of “Group” [F_1,25_ = 8.28; p = 0.0081], “Time” [F_4,100_ = 12.88; p < 0.0001] and “Time x Group” interaction [F_4,100_ = 5.02; p = 0.0014]) (Fig 5C.2).

### Long-term emotional cognition

Repeated testing in the forced swim task during experiment 1 and elevated plus maze during experiment 2 was carried out to collect data related to the offspring’s ability to adaptively habituate to stressful conditions. In experiment 1, the CYP20 offspring’s behavior on day 2 did not show any effects of CYP on immobility time in the forced swim task (Fig 4E.1) and no difference could be observed through minute-by-minute analysis (Fig 4E.2). In CTL and CYP20 offspring, a 24h habituation could be observed as an adaptive increase in immobility time (p = 0.0002 for CTL and p = 0.0022 for CYP20 offspring) (Fig 4E.3). Minute-by-minute analysis showed that this increase occurred for almost the entire time of the test both in CTL and CYP20 offspring (main effects of “Trial” [F_1,38_ = 18.89; p < 0.0001] and “Time” [F_5,190_ = 48.42; p < 0.0001] for the CTL group and main effects of “Trial” [F_1,40_ = 4.68; p = 0.036] and “Time”: F_5,200_ = 46.2; p < 0.0001) (Fig 4E.4. In our second experiment, data showed that CYP5 offspring displayed similar ambulatory exploration to their CTL counterparts on day 2 (Fig 5D.1 and 5D.2). No habituation was observed between day 1 and day 2 in the 2 groups (Fig 5D.3 and 5D.4). Similarly, CYP5 did not have any effect on spatial exploration of the apparatus on day 2 (Fig 5D.5. CTL offspring re-tested in the same apparatus 24h later significantly increased their naturalistic aversion for anxiogenic open arms which was demonstrated by the decreased time spent in open arms (p < 0.0001) and center (p = 0.019), and the increased time spent in closed arms (p = 0.0009) (Fig 5D.6. CYP5 offspring also decreased their open arms (p = 0.0013) and center (p = 0.016) frequentation and increased their frequentation of closed arms (p = 0.0006) (Fig 5D.6, indicating that CYP5 did not affect their ability for spatial exploration.

### Social motivation and social cognition

**Fig 6:**
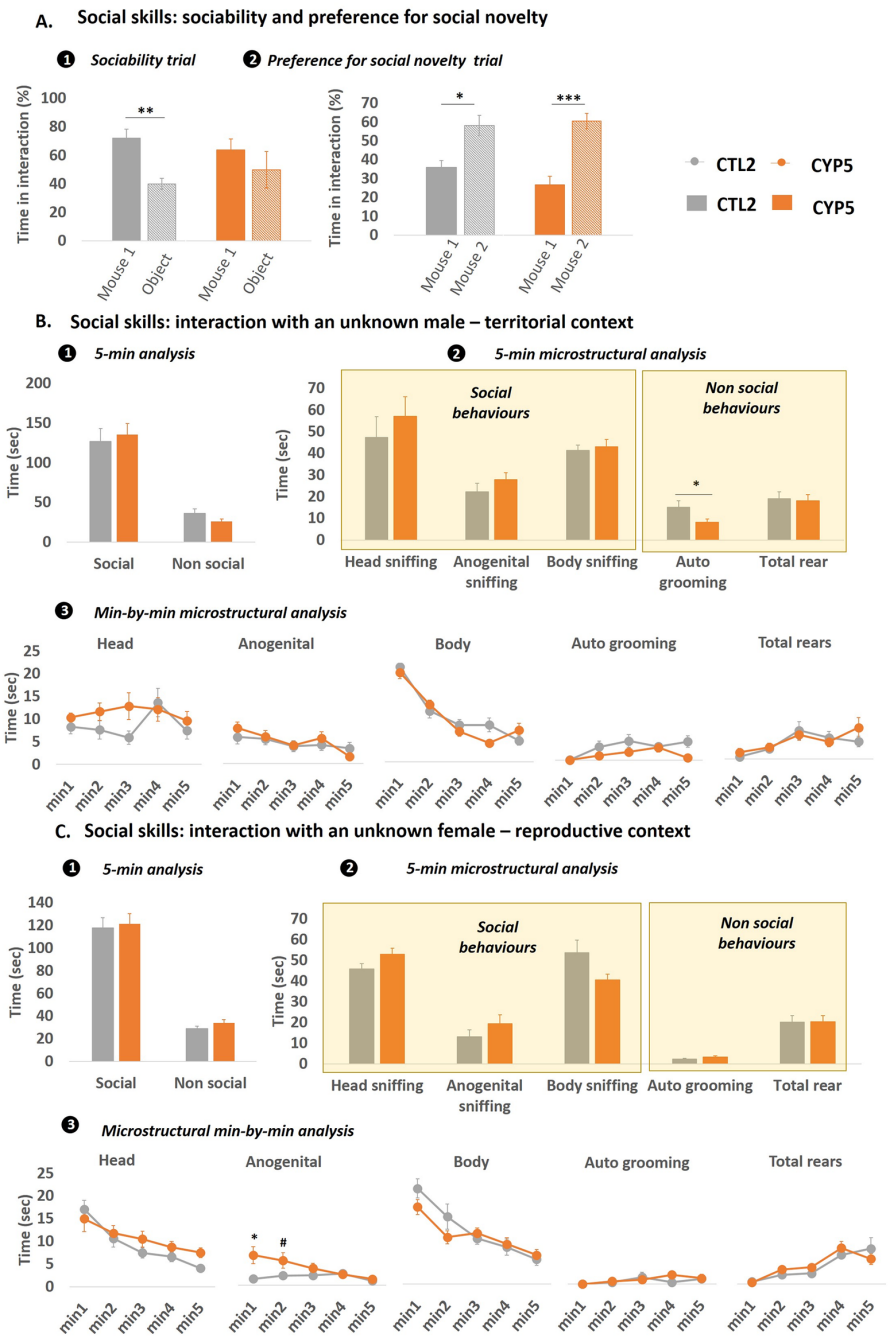
Adult CYP5-exposed offspring displayed maladaptive behavior in response to social contexts. **A**: In the three-chambered sociability test, CYP5-exposed offspring displayed no preference for the chamber containing the social partner (mouse 1), unlike the controls (1). When the object was replaced by a novel social partner (mouse 2) in a subsequent trial, CYP5-exposed offspring, as well as the controls, preferred interacting with the novel partner compared to the already known partner (2). * *p* < 0.05, ** *p* < 0.01, *** *p* < 0.001 compared to time spent in contact with wire cup containing mouse 1. **B**: Social skills were also assessed in more realistic conditions: in a territorial context *i*.*e*. a male—male interaction task and in a **C:** reproductive context *i*.*e*. male—female interaction task. In these tasks, both social and non-social behaviors were scored over the total duration of the test (1). The microstructure of social / non-social behaviors was also monitored (2). And each parameter was measured on a minute-by-minute basis (3). Data are mean +/- sem; n = 13–14 / group. *p < 0.05, ** p < 0.01, *** p < 0.001, # p < 0.09 compared to the controls.

Given the crucial role of sexual hormones in driving the social behavior of mice and given that CYP has been previously shown to disturb the metabolism of sexual hormones in adult male mice after maternal exposure [16], the question of whether their social skills were disturbed was addressed. Offspring were first confronted with the three-chambered sociability task assessing both sociability and preference for social novelty. Data indicated that CTL offspring spent more time close to the cup containing another mouse compared to the cup containing an object (p = 0.0077), while CYP5-exposed offspring did not (Fig 6A.1. However, during the preference for social novelty trial, the two groups displayed significant preference for the novel mouse (p = 0.019 for CTL and p = 0.0009 for CYP5 offspring) (Fig 6A.2. Then test mice were exposed to an unknown male conspecific and their subsequent behavioral response monitored. The 5-min analysis revealed no difference between CYP5 and CTL offspring as the former spent so much time interacting with the intruder than the CTL group (Fig 6B.1. When microstructure of social behavior was taken into account, no effect of CYP was observed. However, CYP5 offspring spent less time expressing auto grooming compared to CTL offspring (Fig 6B.2. Minute-by-minute analysis provided no additional data on disturbing effects of CYP (Fig 6B.3. When exposed to an unknown female, the 5 min analysis did not reveal any effect of CYP5 on the offspring’s social and non-social behaviors (Fig 6C.1 and 6C.2). However, minute-by-minute analysis showed that CYP5 offspring spent significantly more time performing anogenital sniffing than their CTL counterparts at the beginning of the encounter (main effects of “Group” [F_1,125_ = 13.63; p = 0.0003], “Time” [F_4,125_ = 3.35; p = 0.012] and “Group x Time” interaction [F_4,125_ = 2.97; p = 0.022]) (Fig 6C.3.

## Discussion

The present study reports that perinatal exposure to low dose CYP through the inhalation route led to abnormal brain development during the offspring’s early life with behavioral changes lasting until adulthood. CYP is already known to have neurotoxic and negative developmental consequences [8] but little is known about its ability to induce long lasting behavioral defects from the perspective of the DOHAD hypothesis. Despite the fact that several recent studies have investigated enduring cellular and molecular brain consequences of early life exposure to CYP [30, 31], none of them have entirely met the requirements emitted by the OECD guidelines for developmental neurotoxicity testing of chemicals [11]. In agreement with these guidelines, we assessed (1) general toxicity in pregnant / lactating dams, (2) physical and sensory motor development during the early postnatal period in the offspring, (3) whole brain transcriptomic changes in the offspring at the end of the exposure, (4) morphological parameters such as brain and testis weight changes in the offspring at the end of the exposure, and (5) emotional, cognitive and social skills during offspring’s adulthood.

### CYP disturbs behavioral ontogeny in the offspring

Our experiments revealed that maternal physiology and/or behavior are negatively impacted by CYP exposure. This result is in accordance with a recent study conducted in mice which showed a robust decrease of gestational weight after dams were given 10 mg/kg/day cypermethrin in corn oil orally for 4 weeks before mating [32]. However, other studies have shown no such effects at doses similar to ours [33] and even at higher doses of CYP [34, 35]. Such discrepancies may be due to differences in dosing protocol, mouse strain, route of exposure but also, and more probably, to stereoisomer composition of the test compound (75:25 trans-cis vs 60:40 trans-cis in the present study) as insecticidal and toxic effects of CYP (and all other pyrethroids) are known to be highly stereospecific [8]. To our knowledge, no other study has explored maternal welfare/behavior despite the fact that such effects may have significant consequences on the behavior of offspring later in life. The close relationship between maternal behavior during early life and adulthood mental health is now clearly demonstrated whether it be in humans, primates or rodents, especially in terms of emotional functions and endocrine response to stress [36]. Consequently, we cannot preclude the possibility that CYP-induced behavioral alterations reported in adult offspring may result, at least partially, from changes in maternal physiology and/or behavior. Further experiments using lower doses, with no maternal effects, would be necessary to accurately conclude on that point.

Contrary to behavioral outcomes, developmental landmarks and sensory motor functions are not known to be subject of maternal behavioral influences, and hence, enable an assessment of the direct effect of CYP on brain maturation. Our data show that the 2 doses of CYP have negative impacts on the developing brain, especially on motor functions as demonstrated by delayed righting reflex, negative geotaxis and bar grasping. Interestingly, some disturbances seem to be dose-specific such as eyelid opening, delayed only in CYP5 offspring, and auditory startle reflex, delayed only in CYP20 offspring. Overall, these results are in agreement with previous studies showing retardation in righting reflex acquisition, eye opening and pinna detachment in rat offspring prenatally exposed to 15 mg/kg/day CYP [37]. Sensory development is far less affected as demonstrated by the lack of an adverse effect of CYP on communicative skills in response to isolation from the mother. In pups, this ability is strongly dependent of olfactory system integrity [38]. Good olfactory skills are thus essential for pups to correctly respond to olfactory changes in the environment. Therefore, the fact that CYP exposure did not modify communicative skills in pups throughout the overall postnatal period shows that olfactory system development is not altered. At first sight, this could be contrasting with transcriptomic data showing CYP-induced abnormal expression of several olfactory receptor genes. However, many of these olfactory receptors are widely distributed in several non-chemosensory organs in mammals where they play distinct functions not directed to the perception of odors [39]. Development of the auditory system seems to be marginally disturbed as only the highest dose of CYP induced a 1 day delay in the acquisition of the auditory startle reflex. Altogether, our data indicates that CYP-induced neurodevelopmental changes are not severe but rather affect specific processes such as neural systems underlying neuromotor development. Morphological data collected at the end of the dosing period are also in line with non-burdensome effects of CYP as they revealed no changes in brain weight.

### Perinatal exposure to CYP induces long lasting disturbances in offspring’s emotional adaptation to stressful environments and social motivation

#### CYP affects specific aspects of offspring’s emotional cognition

Our behavioral analyses reveal that perinatal exposure to the 2 doses of CYP modified the way offspring cope with stress inherent to testing conditions. These findings are in line with other studies reporting that neonatal exposure to CYP during the highly sensitive period of brain growth spurt (BGS) induces enduring changes in the offspring’s ability to adapt to stressful environments [40]. According to these authors, these alterations are likely to be irreversible as adverse effects occurred in both 2 month old and 5 month old male mice. Other pyrethroids have been shown to induce long-lasting behavioral abnormalities in exposed offspring such as permethrin which when administered during the BGS period of life causes lifelong deficits in emotional cognition through altered communication between the amygdala (AMY) and the hippocampus (HIPPO) [41], mediated probably by oxidative stress [42]. Interestingly, our findings seem to be reliable as it has been observed in both CYP20 and CYP5 offspring, suggesting that such consequences are not dose-specific. In support of this statement, none of the 2 doses altered the offspring’s behavior in response to weakly challenging environments such as the elevated plus maze and openfield tests. These findings are in agreement with other reports showing a lack of effect of neonatal exposure to pyrethroids on the offspring’s explorative behavior in these two devices [41].

This points to important considerations regarding behavioral toxicology as our data demonstrates the paramount importance of using ethological tools to perform thorough analysis of mice behavior and then reliably characterizing toxicity of hazardous compounds. Indeed, confronting mice to escapable and inescapable conditions does not inform the same processes as they lead to different behavioral outputs, each with specific underlying neural substrates [43]. By separating escapable and inescapable conditions on the one hand, and low *vs* high light level on the other hand, our experimental design was likely to detect that CYP induced deleterious emotional effects only in conditions generating high levels of stress. Time analysis substantiates our hypothesis of a specific effect of CYP on offspring’s behavior by showing that CYP-induced behavioral defects are both context and time-dependent. Indeed, if we consider the forced swim task, both CYP20 and CYP5 offspring displayed a higher immobility time, especially at the end of the test. Albeit to a lesser extent, this was also true in the tail suspension task. However, if we consider the 10 min challenge in a new cage, only early behavioral response seemed to be affected while short-term habituation skills remained undisturbed. This indicates that both immediate response to stress and short-term habituation to stress are negatively impacted by CYP and that these alterations are context-specific. This is not so surprising given that different stressors, depending on their inherent qualitative characteristics (intensity, duration) are known to activate different components of the stress system [44], the response to a physiological stressor requiring the involvement of only hypothalamus and brainstem whereas structures such as the AMY or HIPPO and prefrontal cortex (PFC) are recruited to respond to somewhat more complex environmental conditions [45]. If emotional cognition was assessed as it is made in classical neurotoxicity studies *i*.*e*. through the use of the sole elevated plus maze or openfield tests for instance, our conclusion would have been that CYP did not exert any negative effects on the offspring’s ability to cope with stressful conditions, which is clearly not the case. Such results support the importance for behavioral scientists not only to vary testing conditions but also to study dynamics of behavior when assessing for toxicity, even if this approach is more and more common in behavioral testing protocols [14, 46, 47].

Long-term habituation to emotional tasks was also addressed in our study by re-testing mice in already known apparatuses and it seems to be unaltered by CYP exposure as demonstrated by the lack of deleterious effects during the 24h retest trial in the forced swim task and the elevated plus maze. This means that the capacity of CYP-exposed offspring to keep aversive features inherent to testing conditions in memory is preserved despite their inability to correctly adapt their behavior during the first trial of the task. However, the main drawback in our design is related to the fact that long-term habituation of CYP5 offspring was evaluated in the elevated plus maze (in weakly emotional conditions) while in CYP20 offspring it was assessed in the forced swim task (a highly challenging condition). Therefore, we cannot rule out the possibility that CYP5 offspring would have behaved differently if tested in a highly challenging condition. This has to be addressed in order to make a confident conclusion. Additionally, the finding does not preclude the possibility for other type of memory to be affected by CYP as demonstrated in other works in which the type II pyrethroids disturbed working memory in rats after neonatal exposure [48].

#### CYP affects specific aspects of offspring’s social behavior

Social behavior of offspring has also been explored in the current study. To our knowledge, this is the first work assessing this behavioral domain in response to developmental exposure to pyrethroids. In accordance with our strategy to obtain a maximum of information in a limited number of tests, we used two types of behavioral tests, each associated with a different level of reinforcement. The reader needs to keep in mind that the expression of behavior is closely related to the reinforcing characteristics of a stimulus. In the three chamber apparatus, the stimulus mice were constrained in a compartment that allowed sniffing and interaction but no physical contact *i*.*e*. with limited reinforcements. On the contrary, during the social interaction tests, testing mice were directly confronted to stimulus mice and in this case, both positive and negative reinforcements may occur, leading potentially to quite different behavioral responses from the testing mouse.

Our data indicates that CYP has modified social behavior in adult male offspring as evidenced, first, in the three-chambered sociability test in which CYP5-exposed offspring displayed altered response in the sociability trial while they were normal in the preference for social novelty trial. This finding shows that only some dimensions of social behavior are affected by CYP exposure. Basically, the sociability trial measures social motivation whereas the preference for social novelty trial measures social memory / recognition [49]. Our results therefore show that CYP may disturb social motivation but not social recognition. Of note, processing social motivation and social recognition does not involve the same circuitry in the brain. The former involves reciprocal relationships between the prefrontal cortex (PFC) and striatal areas mediating rewarding aspects of social interaction like the nucleus accumbens (NAC) and the ventral tegmental area (VTA) [50]. The latter rather implicates reciprocal relationships between the PFC and HIPPO or AMY − not surprisingly given the importance of these structures in memory formation [51, 52]. In keeping with this organization of the social circuitry, one can assume that CYP preferentially affects the PFC-NAC-VTA circuitry rather than the PFC-AMY-HIPPO circuitry. Such a hypothesis is consistent with other studies showing that rats treated from PND6 to PND21 with permethrin displayed working memory deficits involving the frontal cortico-striatal circuitry later in their lives [48]. Earlier, the same group demonstrated that neonatal permethrin exposure induced dopamine and Nurr1 reduction associated with higher lipid peroxidation in the striatum attesting to its ability to disturb the frontal cortico-striatal circuitry through oxidative processes [42]. Other studies also provide strong evidence that striatum is a key structure accounting for a substantial part of CYP-related developmental neurotoxicity [53–56]. Deficits in social motivation observed in our study consolidates this hypothesis.

In the more naturalistic condition of a direct dyadic interaction, CYP5-exposed offspring only displayed slight behavioral changes in response to an unknown intruder. When facing a male intruder (informing on territorial defense) CYP5-exposed males were as importantly engaged in social exploration as their CTL counterparts. At first sight, this could be interpreted as being in contradiction to the abovementioned finding, but, as previously stated, the two testing conditions are not equivalent from an ethological point of view. Indeed, during dyadic encounters, physical contacts are allowed and make the situation much more reinforcing (positively or negatively), leading to quite different behavioral response. This result implies that CYP-induced adverse effects are likely to be context specific, strengthening our statement to perform ethological analyses by conducting behavioral testing under varying contextual conditions. Interestingly, CYP5-exposed offspring spent less time emitting auto grooming, indicating an inability to correctly adapt to the emotional value generated by the social situation. Such a disturbance has been described to inform on social anxiety and has been associated with abnormal functioning of cerebral structures such as the AMY and the striatum (STR), controlling respectively emotion and motor-related field of self-grooming behavior [57]. Our results also indicate that mating behavior may be affected as CYP5-exposed offspring displayed maladaptive patterning of anogenital sniffing, with an increased rate of sniffing at the beginning of the test followed by habituation while CTL offspring displayed constant anogenital sniffing throughout the test. Such a difference may reflect disturbances in coping strategy during male-female social encounter. Additionally, this finding stresses that social behavior needs to be more thoroughly and dynamically investigated in order to refine data interpretation through the collection of more reliable information. Indeed, during such laboratory situation, the one or two first minutes of the test are particularly relevant from an ethological point of view as they are more “emotionally” and “socially” charged. Regarding the effect of CYP on this parameter, it is difficult to go deeper in our discussion as, to our knowledge, there is only one study addressing the question of whether early exposure to CYP might disturb social behavior in the offspring [32]. However, the study design was very different from ours as CYP exposure occurred before mating only and social behavior was measured in 1 month old pups. Despite such discrepancies, Farag and collaborators reported a reduced time spent in active social interaction (10 mg/kg/day CYP in corn oil orally for 4 weeks/5 days in a week before parents’ mating). To our knowledge, this is thus the first study addressing social disturbances in response to perinatal exposure to cypermethrin, and more largely to pyrethroids. Even if our data does not reflect any massive effect of CYP on social abilities in the context of social interaction, it suggests that fine-tuned processes might be affected. As a consequence, CYP-mediated changes in social behavior need to be addressed more thoroughly by using adapted testing procedures and testing conditions assessing the multiple dimensions of mice social behavior.

### Mitochondrial dysfunction as candidate mechanism for CYP-induced neurodevelopmental and behavioral alterations

Transcriptomic data collected in the current study shows that the majority of genes found to be differentially expressed between CTL1 and CYP20 offspring on the one hand and between CTL2 and CYP5 offspring on the other hand, are involved in mitochondrial functioning. Even if the set of dysregulated genes is not strictly similar depending on the dose, the expression of some genes seems to be commonly affected by the 2 doses of CYP: this is the case for *Cox7c*, *Timm8a1*, *Mertk*, *Rps27a* and *Hamp*. Remarkably, 2 of these 5 genes are known to be importantly involved in mitochondrial homeostasis. The first one, *Cox7c*, encodes a subunit of the cytochrome c oxidase enzyme (COX), also known as mitochondrial complex IV, which mediates the terminal step of the mitochondrial electron transport chain. *Cox7c* is one of the 13 sub-units constituting the COX complex [58]. It has been shown to play a critical role in the correct assembly of the nuclear-encoded sub-units forming the COX complex [59]. COX deficiency has been associated with developmental defects and neurodegeneration in a zebra fish model [60] and motor dysfunctions in a drosophila model [61]. CYP exposure also resulted in *Timm8a1* upregulation. This gene encodes the translocase of inner mitochondrial membrane 8 belonging to a complex of proteins found in the inner membrane of the mitochondria. The functional role of this complex is to transport to the mitochondria membrane all the proteins produced from nuclear DNA that are needed to build and maintain a functioning mitochondria [62]. Disturbances of such a mechanism may significantly impair the organelle’s ability to produce energy and can thus have disastrous consequences, not only due to the primary loss of ATP or indirect impairment of “downstream” functions but also due to increased reactive oxygen species (ROS) production. Such an assumption is in line with what is already known regarding CYP toxicity. Indeed, strong evidence of the involvement of oxidative stress processes in neurotoxic and reprotoxic properties of CYP are provided in the literature. This has been demonstrated *in vitro* [63–65] and *in vivo* in several species such as rats, mice, rabbits, zebra fish, carp, mussels, crayfish [66–71]. CYP has also been shown to induce apoptosis in rat astrocyte through an elevated generation of ROS [72]. It has also been demonstrated *in vivo* that CYP causes oxidative stress-mediated neurotoxicity in rats in association with increased ROS production [73]. According to these studies and others, CYP-induced oxidative damage is likely to originate from alterations in the antioxidant defense system and mitochondrial dysfunctions leading to high levels of ROS and increased apoptosis [56, 74]. Given these toxic features of CYP, transcriptional profiling was analyzed through pathway-focused real-time PCR arrays and it has been demonstrated that CYP disturbed transcript levels of several genes involved in DNA replication and repair, apoptosis, cell cycle, oxidative stress, and toxicity pathways [75]. To some extent, these findings corroborate our transcriptomic data and enable us to assume that mitochondrial dysfunctions might be due to increased CYP-related production of ROS. Even if we cannot bring any direct evidence of such a defect, this assumption is in agreement with the overall literature demonstrating that ROS-induced oxidative stress is a central hallmark of CYP-induced toxicity, whether it be in the brain or in other tissue such as testis [76]. Proteins being among the main targets for oxidants mainly due to their abundance in biological systems [77], it would not be surprising to observe proteostasis alterations in CYP-exposed offspring. Interestingly, several genes related to protein synthesis (*Rps27a*, *Rps18*, *Rps16*, *Rpl13*, *Rpl15*, *bRpl35*, *Rpl37*, *Rpl9*, *Bambi*, *Mrpl14*, *Taf7*), protein maturation (*C1galt1c1*, *Acer2*, *Nanp*, *β3Galt4*) and protein degradation (*Usmg5*, *Nr1d1*, *Otub2*, *Iqub*, *Gsp*, *Magea9*, *Ppt1*, *Plekhf1*, *Clnd5*, *Pbk*, *Usp-ps*) were found to be dysregulated in either CYP20 or CYP5 offspring, clearly substantiating such a hypothesis. However, such an assumption deserves to be explored more thoroughly.

In recent years, increasing evidence has shown that alterations in proteostasis and mitochondrial dysfunction are accompanying neurodegenerative diseases such as Huntington's disease, Alzheimer's disease and Parkinson's disease (PD) [78]. Interestingly, CYP exposure has been associated with pathological features strikingly reminiscent of PD symptoms [30, 55]. Considering the transcriptomic data reported in the present study, our results support this assumption. Our behavioral data is also in accordance with a CYP-induced PD-like syndrome as behavioral changes reported here are classically observed in animal models of PD, particularly in the premotor stage of PD syndrome [79]. Indeed, although PD is classically considered as a movement disorder, an increasing body of evidence suggests that PD patients and parkinsonian animals also display a variety of non-motor symptoms affecting the emotional and cognitive dimensions of behavior [80]. In support of this, several studies have reported that parkinsonian mice display maladaptive behaviors in the forced swim task and the tail suspension task similar to those we observed in the present study [81, 82]. Another study has found similar results in rats in a pre-motor stage model of PD [79].

### Concluding remarks: Towards an AOP relevant to developmental neurotoxicity of type II pyrethroids

**Fig 7:**
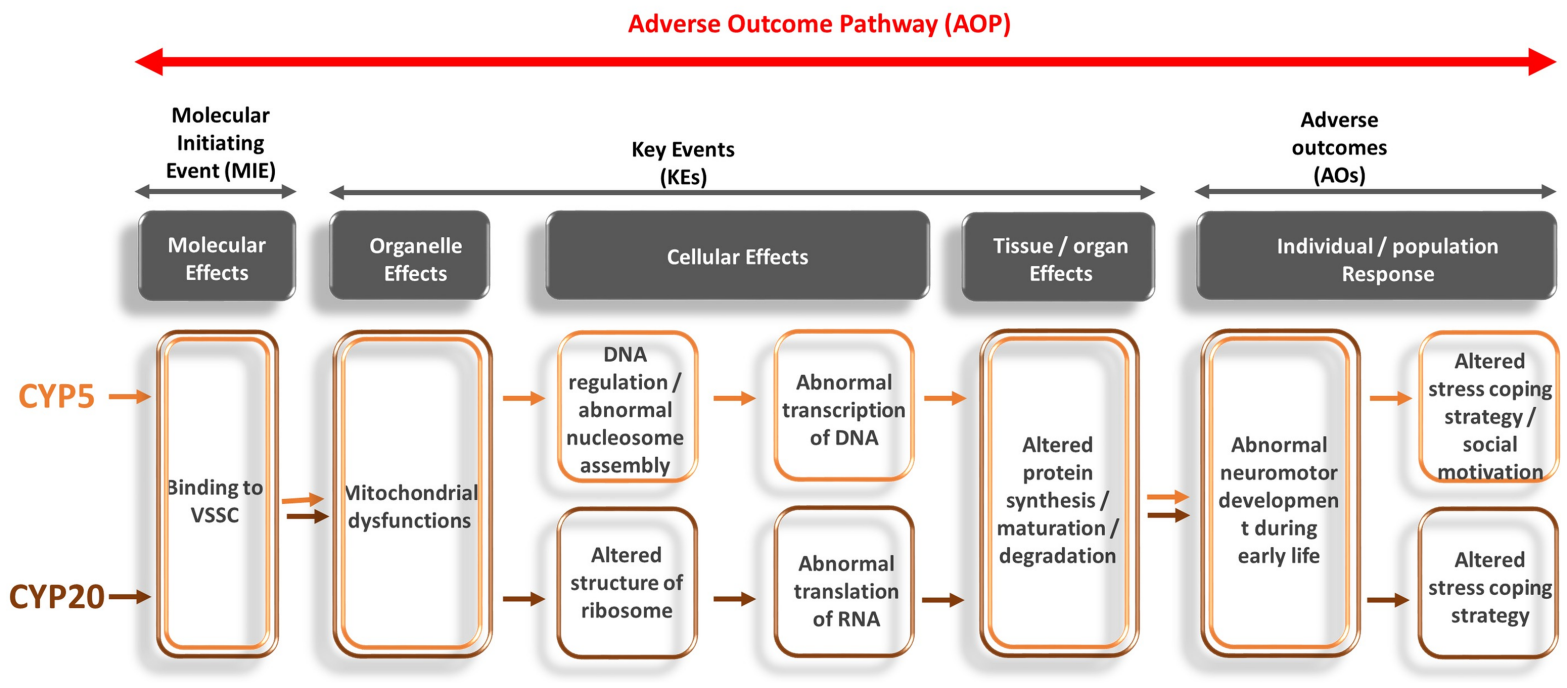
Putative Adverse Outcome Pathway for CYP-induced developmental neurotoxicity. Brown boxes show molecular initiating events (MIE), key events (KEs) and adverse outcomes (AOs) related to cypermethrin exposure at the highest dose (20mg/kg; CYP20). Orange boxes show MIE, KEs and AOs related to cypermethrin exposure at the lower dose (5mg/kg; CYP5). Common mechanisms / commonly observed changes are in double-lined boxes.

By showing that *in utero* and lactational exposure to the pyrethroid insecticide cypermethrin induces abnormal brain development during early life, long-lasting behavioral defects during adulthood and gene expression changes related to mitochondrial functioning, DNA regulation and protein metabolism, the present study strongly substantiates the DOHAD hypothesis supported by many authors including [83, 84]. In line with this, data collected in the present study raises fundamental question on developmental neurotoxicity safety assessment procedures in guidelines emitted by the US EPA and the OECD. Indeed, these guidelines only recommend addressing learning and memory when testing for long-term effects of early exposure to chemicals [11]. However, we cannot preclude the possibility that social and / or emotional dimensions of behavior may be altered whereas learning and memory do not. To some extent, this is what we observed in our study. Therefore, our findings emphasize the importance of using refined behavioral protocols to comprehensively, and above all, multidimensionally, characterize long-term neurotoxic effects of developmental exposure to environmental toxins. Additionally, data provided in the present study can be of particular value in view of the recently implemented Adverse Outcome Pathway (AOP) concept which has been proposed to support a paradigm shift in regulatory toxicology testing and risk assessment [85]. The AOP approach describes a molecular initiating event (MIE) triggering a series of measurable key events (KEs; *i*.*e*. cellular, anatomical, and/or functional changes in biological processes) linking chemical exposure to an adverse outcome (AO) at the organism level. Considering behavioral and transcriptomic data collected in the present study, we propose an AOP relevant to developmental neurotoxicity of cypermethrin and other type II pyrethroid compounds (Fig 7) including “regulation of DNA transcription”, “protein metabolism”, and “mitochondrial defects” as KEs of importance. Although this AOP is not fully described, it could serve as a basis for further development.

## Supporting information

S1 Table DNA microarray analysis: Significantly dysregulated gene transcripts in the brain of CYP20-exposed offspring. (DOCX)

S2 Table DNA microarray analysis: Significantly dysregulated gene transcripts in the brain of CYP5-exposed offspring. (DOCX)

## Abbreviations

CYP: cypermethrin
DNT: Developmental Neurotoxicity Testing
DOHAD: Developmental Origins of Health and Diseases
NOAEL: No Observable Adverse Effect Level
OECD: European Organization for Economic Cooperation and Development
VSSC: voltage-sensitive sodium channels
