# Supplementary material for: In utero and lactational exposure to low-doses of the pyrethroid insecticide cypermethrin leads to neurodevelopmental defects in male mice—An ethological and transcriptomic study
Source: PLoS One. 2017 Oct 11;12(10):e0184475. doi: 10.1371/journal.pone.0184475 (PMC5636066; doi:10.1371/journal.pone.0184475)
Supplement: Supplementary file 1 [file pone.0184475.s001.docx]

S1 table. DNA microarray analysis: significantly dysregulated gene transcripts in the brain of CYP20-exposed offspring

Transcripts Cluster Id	FC (abs) ([Ctrl1] vs [Cyp20])	FC ([Ctrl1] vs [Cyp20])	Log FC ([Ctrl1] vs [Cyp20])	Regulation ([Ctrl1] vs [Cyp20])	Gene Description	Gene Symbol
6950254	1.50	-1.50	-0.58	**down**	taste receptor, type 2, member 102	Tas2r102
6755306	1.47	-1.47	-0.55	**down**	olfactory receptor 420	Olfr420
6907255	1.42	-1.42	-0.51	**down**	histone cluster 2, H4	Hist2h4
6939968	1.39	-1.39	-0.47	**down**	ribosomal protein L13	Rpl13
6963101	1.37	-1.37	-0.45	**down**	olfactory receptor 599	Olfr599
6873475	1.36	-1.36	-0.45	**down**	upregulated during skeletal muscle growth 5	Usmg5
6888680	1.35	-1.35	-0.44	**down**	olfactory receptor 32	Olfr32
6933818	1.35	-1.35	-0.44	**down**	harakiri, BCL2 interacting protein (contains only BH3 domain)	Hrk
6789754	1.32	-1.32	-0.40	**down**	cytochrome c oxidase, subunit VIIc | cytochrome c oxidase, subunit VIIc pseudogene	Cox7c|Gm10012
6949693	1.32	-1.32	-0.40	**down**	ribosomal protein S27A	Rps27a
6870964	1.32	-1.32	-0.40	**down**	RIKEN cDNA 4833408A19 gene	4833408A19Rik
6994751	1.30	-1.30	-0.38	**down**	olfactory receptor 150 | olfactory receptor 919	Olfr150|Olfr919
6811684	1.29	-1.29	-0.37	**down**	histone cluster 1, H2bf	Hist1h2bf
6878761	1.28	-1.28	-0.36	**down**	olfactory receptor 1009	Olfr1009
6847226	1.28	-1.28	-0.36	**down**	Finkel-Biskis-Reilly murine sarcoma virus (FBR-MuSV) ubiquitously expressed (fox derived)	Fau
6985102	1.28	-1.28	-0.36	**down**	ribosomal protein S18	Rps18
6954693	1.28	-1.28	-0.36	**down**	RIKEN cDNA 4931417E11 gene	4931417E11Rik
6819570	1.27	-1.27	-0.35	**down**	C1q and tumor necrosis factor related protein 9	C1qtnf9
6820088	1.27	-1.27	-0.34	**down**	nudix (nucleoside diphosphate linked moiety X)-type motif 18	Nudt18
6791282	1.26	-1.26	-0.33	**down**	nuclear receptor subfamily 1, group D, member 1 | thyroid hormone receptor alpha	Nr1d1|Thra
6945821	1.26	-1.26	-0.33	**down**	olfactory receptor 447	Olfr447
6986741	1.26	-1.26	-0.33	**down**	ribosomal protein L29	Rpl29
7013954	1.25	-1.25	-0.32	**down**	armadillo repeat containing, X-linked 5	Armcx5
6881439	1.25	-1.25	-0.32	**down**	ribosomal protein L29 | predicted gene 3550	Rpl29|Gm3550
6972159	1.24	-1.24	-0.31	**down**	cytochrome c oxidase, subunit VIIIb	Cox8b
6819275	1.23	-1.23	-0.30	**down**	leukotriene B4 receptor 2	Ltb4r2
6779839	1.23	-1.23	-0.30	**down**	hemoglobin alpha, adult chain 1 | hemoglobin alpha, adult chain 2	Hba-a1|Hba-a2
6927800	1.23	-1.23	-0.29	**down**		
6796561	1.23	-1.23	-0.29	**down**	acyl-CoA thioesterase 2 | acyl-CoA thioesterase 1	Acot2|Acot1
6850661	1.22	-1.22	-0.28	**down**	mitochondrial ribosomal protein L14	Mrpl14
6805381	1.21	-1.21	-0.27	**down**	histone cluster 1, H1c	Hist1h1c
6888531	1.21	-1.21	-0.27	**down**	olfactory receptor 1155	Olfr1155
6918178	1.21	-1.21	-0.27	**down**	RIKEN cDNA B330016D10 gene	B330016D10Rik
6824146	1.20	-1.20	-0.27	**down**	RIKEN cDNA 5730469M10 gene	5730469M10Rik
6797538	1.20	1.20	0.26	**up**	OTU domain, ubiquitin aldehyde binding 2	Otub2
6880629	1.20	1.20	0.27	**up**	adenosine deaminase-like	Adal
6975443	1.20	1.20	0.27	**up**	RIKEN cDNA 6430573F11 gene	6430573F11Rik
6952167	1.20	1.20	0.27	**up**	IQ motif and ubiquitin domain containing	Iqub
6990564	1.20	1.20	0.27	**up**	LysM, putative peptidoglycan-binding, domain containing 2	Lysmd2
7003699	1.21	1.21	0.27	**up**	zinc finger protein 709 | RIKEN cDNA C130026I21 gene	Zfp709|C130026I21Rik
6861639	1.21	1.21	0.27	**up**	gastrin releasing peptide	Grp
7020542	1.21	1.21	0.27	**up**	gap junction protein, alpha 6	Gja6
6904367	1.21	1.21	0.27	**up**	predicted gene 5148	Gm5148
6881087	1.21	1.21	0.27	**up**	c-mer proto-oncogene tyrosine kinase	Mertk
6926871	1.21	1.21	0.28	**up**	coiled-coil-helix-coiled-coil-helix domain containing 2	Chchd2
6917219	1.21	1.21	0.28	**up**		BC003266
6766287	1.21	1.21	0.28	**up**	NHS-like 1	Nhsl1
7014799	1.21	1.21	0.28	**up**		
6835403	1.21	1.21	0.28	**up**	transmembrane protein 74	Tmem74
7015995	1.21	1.21	0.28	**up**	zinc finger protein 300	Zfp300
6965558	1.22	1.22	0.28	**up**	vomeronasal 1 receptor 84	Vmn1r84
6940534	1.22	1.22	0.29	**up**	mitogen-activated protein kinase 10	Mapk10
6833186	1.23	1.23	0.29	**up**	RIKEN cDNA 2310016M24 gene	2310016M24Rik
6966327	1.23	1.23	0.30	**up**	hepcidin antimicrobial peptide | hepcidin antimicrobial peptide 2	Hamp|Hamp2
6858465	1.23	1.23	0.30	**up**	BMP and activin membrane-bound inhibitor, homolog (Xenopus laevis)	Bambi
6941248	1.23	1.23	0.30	**up**	ribosomal protein L37	Rpl37
6760339	1.23	1.23	0.30	**up**	zinc finger protein 617	Zfp617
6763941	1.23	1.23	0.30	**up**		
7016427	1.24	1.24	0.31	**up**	C1GALT1-specific chaperone 1	C1galt1c1
6983076	1.24	1.24	0.31	**up**	RIKEN cDNA 9530019H20 gene	9530019H20Rik
7021355	1.24	1.24	0.31	**up**	ribosomal protein L35 | predicted gene 10269	Rpl35|Gm10269
6864747	1.24	1.24	0.31	**up**	TAF7 RNA polymerase II, TATA box binding protein (TBP)-associated factor	Taf7
6818920	1.24	1.24	0.32	**up**	olfactory receptor 741	Olfr741
6916219	1.25	1.25	0.32	**up**	KTI12 homolog, chromatin associated (S. cerevisiae)	Kti12
6996110	1.26	1.26	0.33	**up**	SUMO/sentrin specific peptidase 8	Senp8
7007744	1.26	1.26	0.33	**up**	defensin, alpha, 25 | spermatogenesis associated glutamate (E)-rich protein 4a | RIKEN cDNA 5031410I06 gene | predicted gene 10220 | predicted gene 4301 | predicted gene 4312 | vomeronasal 2, receptor, pseudogene 11 | defensin, alpha, pseudogene 1 | predicted gene 6696 | predicted pseudogene 15299 | predicted gene 10471 | vomeronasal 2, receptor 7 | predicted gene 7361 | predicted gene 5862 | predicted gene 7347	Defa25|Speer4a|5031410I06Rik|Gm10220|Gm4301|Gm4312|Vmn2r-ps11|Defa-ps1|Gm6696|Gm15299|Gm10471|Vmn2r7|Gm7361|Gm5862|Gm7347
6832105	1.26	1.26	0.33	**up**	X-prolyl aminopeptidase (aminopeptidase P) 3, putative	Xpnpep3
6935917	1.26	1.26	0.34	**up**	mitochondrial transcription termination factor | predicted gene 9897	Mterf|Gm9897
6793326	1.27	1.27	0.34	**up**	retinol dehydrogenase 14 (all-trans and 9-cis)	Rdh14
6879185	1.27	1.27	0.35	**up**		
6765982	1.29	1.29	0.36	**up**	translocase of inner mitochondrial membrane 8 homolog a1 (yeast)	Timm8a1
6965111	1.29	1.29	0.36	**up**	olfactory receptor 539	Olfr539
6776875	1.29	1.29	0.36	**up**	predicted gene 5136	Gm5136
6788133	1.29	1.29	0.37	**up**	RIKEN cDNA 0610009B22 gene	0610009B22Rik
6981447	1.29	1.29	0.37	**up**	testis expressed gene 24	Tex24
6840199	1.30	1.30	0.38	**up**	LIM domain containing preferred translocation partner in lipoma	Lpp
6924813	1.31	1.31	0.39	**up**	4-hydroxyphenylpyruvate dioxygenase-like	Hpdl
6780732	1.32	1.32	0.40	**up**	olfactory receptor 10	Olfr10
6924922	1.34	1.34	0.42	**up**	olfactory receptor 1338	Olfr1338
6911576	1.34	1.34	0.43	**up**	RIKEN cDNA D130060J02 gene	D130060J02Rik
6817137	1.36	1.36	0.44	**up**	RIKEN cDNA 5430414B19 gene	5430414B19Rik
6970084	1.36	1.36	0.45	**up**	olfactory receptor 666	Olfr666
6868909	1.50	1.50	0.58	**up**	RIKEN cDNA C030016D13 gene	C030016D13Rik
