# Supplementary material for: In utero and lactational exposure to low-doses of the pyrethroid insecticide cypermethrin leads to neurodevelopmental defects in male mice—An ethological and transcriptomic study
Source: PLoS One. 2017 Oct 11;12(10):e0184475. doi: 10.1371/journal.pone.0184475 (PMC5636066; doi:10.1371/journal.pone.0184475)
Supplement: Supplementary file 2 [file pone.0184475.s002.docx]

S2 table. DNA microarray analysis: significantly dysregulated gene transcripts in the brain of CYP5-exposed offspring

Transcripts Cluster Id	FC (abs) ([Ctrl2] vs [Cyp5])	FC ([Ctrl2] vs [Cyp5])	Log FC ([Ctrl2] vs [Cyp5])	Regulation ([Ctrl2] vs [Cyp5])	Gene Description	Gene Symbol
6887972	1.20	-1.20	-0.26	**down**	tetratricopeptide repeat domain 30B	Ttc30b
6915138	1.20	-1.20	-0.27	**down**	alkaline ceramidase 2	Acer2
6894345	1.20	-1.20	-0.27	**down**	olfactory receptor 1138	Olfr1138
6917651	1.21	-1.21	-0.28	**down**	predicted gene 5589	Gm5589
6872661	1.22	-1.22	-0.28	**down**	ribosomal protein L9	Rpl9
6766373	1.22	-1.22	-0.29	**down**	predicted gene 6251	Gm6251
6853079	1.22	-1.22	-0.29	**down**	N-acetylneuraminic acid phosphatase	Nanp
6769607	1.22	-1.22	-0.29	**down**	pro-melanin-concentrating hormone | RIKEN cDNA 4930547N16 gene	Pmch|4930547N16Rik
6854604	1.22	-1.22	-0.29	**down**	FK506 binding protein 5	Fkbp5
6916915	1.23	-1.23	-0.30	**down**	palmitoyl-protein thioesterase 1	Ppt1
6811678	1.24	-1.24	-0.31	**down**	histone cluster 1, H4a | histone cluster 1, H4b | histone cluster 1, H4c | histone cluster 1, H4f | histone cluster 1, H4m | predicted gene 11275	Hist1h4a|Hist1h4b|Hist1h4c|Hist1h4f|Hist1h4m|Gm11275
6761691	1.24	-1.24	-0.32	**down**	diazepam binding inhibitor	Dbi
6971280	1.25	-1.25	-0.32	**down**	sulfotransferase family 1A, phenol-preferring, member 1	Sult1a1
6881087	1.25	-1.25	-0.32	**down**	c-mer proto-oncogene tyrosine kinase	Mertk
6949693	1.25	-1.25	-0.32	**down**	ribosomal protein S27A	Rps27a
6957050	1.25	-1.25	-0.33	**down**	ribosomal protein L15	Rpl15
6978290	1.26	-1.26	-0.34	**down**	metallothionein 2	Mt2
6942133	1.27	-1.27	-0.34	**down**	ribosomal protein S16 | ribosomal protein S16, pseudogene 2	Rps16|Rps16-ps2
6900970	1.27	-1.27	-0.35	**down**	coagulation factor III	F3
6966610	1.27	-1.27	-0.35	**down**	pleckstrin homology domain containing, family F (with FYVE domain) member 1	Plekhf1
7021920	1.28	-1.28	-0.35	**down**	spermiogenesis specific transcript on the Y 2 | similar to spermiogenesis specific transcript on the Y 2 | spermiogenesis specific transcript on the Y 2-like | y-linked testis-specific protein 1-like	Ssty2|LOC434960|LOC100040744 LOC100039574|LOC100042428|LOC100039926|LOC100042668
7022934	1.28	-1.28	-0.35	**down**		
6940604	1.28	-1.28	-0.36	**down**		LOC100503439
6970090	1.30	-1.30	-0.37	**down**	olfactory receptor 667	Olfr667
6789754	1.30	-1.30	-0.38	**down**	cytochrome c oxidase, subunit VIIc | cytochrome c oxidase, subunit VIIc pseudogene	Cox7c|Gm10012
7011905	1.32	-1.32	-0.40	**down**	melanoma antigen, family A, 9, pseudogene	Magea9-ps
6949310	1.36	-1.36	-0.44	**down**	RIKEN cDNA 8430408G22 gene	8430408G22Rik
7023358	1.37	-1.37	-0.45	**down**	similar to spermiogenesis specific transcript on the Y 2 | spermiogenesis specific transcript on the Y 2-like	LOC434960|LOC100040744|LOC100042428|LOC100039574
6899760	1.38	-1.38	-0.46	**down**	thioredoxin interacting protein	Txnip
7023433	1.43	-1.43	-0.52	**down**	spermiogenesis specific transcript on the Y 2 | similar to spermiogenesis specific transcript on the Y 2 | spermiogenesis specific transcript on the Y 2-like | y-linked testis-specific protein 1-like	Ssty2|LOC434960|LOC100040744 LOC100039574|LOC100042428|LOC100039926|LOC100042668
6888531	1.48	-1.48	-0.56	**down**	olfactory receptor 1155	Olfr1155
6755306	1.64	-1.64	-0.71	**down**	olfactory receptor 420	Olfr420
6878710	1.20	1.20	0.27	**up**	translocase of inner mitochondrial membrane 10 homolog (yeast)	Timm10
6857797	1.20	1.20	0.27	**up**	zinc finger protein 36, C3H type-like 2	Zfp36l2
6966327	1.21	1.21	0.27	**up**	hepcidin antimicrobial peptide | hepcidin antimicrobial peptide 2	Hamp|Hamp2
6915269	1.21	1.21	0.27	**up**	doublesex and mab-3 related transcription factor like family A1	Dmrta1
6777956	1.21	1.21	0.27	**up**	neurexophilin 4	Nxph4
6917063	1.21	1.21	0.27	**up**	RIKEN cDNA 9930104L06 gene	9930104L06Rik
6929632	1.21	1.21	0.28	**up**	centromere protein A	Cenpa
7011603	1.21	1.21	0.28	**up**	eukaryotic translation initiation factor 4E	Eif4e
6875695	1.22	1.22	0.28	**up**	Notch-regulated ankyrin repeat protein	Nrarp
6811692	1.22	1.22	0.29	**up**	histone cluster 1, H4a | histone cluster 1, H4c | histone cluster 1, H4f	Hist1h4a|Hist1h4c|Hist1h4f
6769111	1.22	1.22	0.29	**up**	olfactory receptor 1351	Olfr1351
6765982	1.22	1.22	0.29	**up**	translocase of inner mitochondrial membrane 8 homolog a1 (yeast)	Timm8a1
6891880	1.23	1.23	0.29	**up**	CD93 antigen	Cd93
7016678	1.23	1.23	0.30	**up**	apelin	Apln
6854985	1.23	1.23	0.30	**up**	UDP-Gal:betaGlcNAc beta 1,3-galactosyltransferase, polypeptide 4	B3galt4
6819883	1.24	1.24	0.31	**up**	PDZ binding kinase	Pbk
6969968	1.26	1.26	0.33	**up**	ubiquitin specific peptidase, pseudogene (USP17 homolog) | deubiquitinating enzyme 2a | ubiquitin specific peptidase 17-like 5	Usp-ps|Dub2a|Usp17l5
6970084	1.27	1.27	0.35	**up**	olfactory receptor 666	Olfr666
6939241	1.28	1.28	0.35	**up**	kinase insert domain protein receptor	Kdr
6972168	1.30	1.30	0.37	**up**	interferon induced transmembrane protein 3	Ifitm3
6878739	1.31	1.31	0.39	**up**	apelin receptor	Aplnr
6839863	1.37	1.37	0.46	**up**	claudin 5	Cldn5
